# AKTIP/Ft1, a New Shelterin-Interacting Factor Required for Telomere Maintenance

**DOI:** 10.1371/journal.pgen.1005167

**Published:** 2015-06-25

**Authors:** Romina Burla, Mariateresa Carcuro, Grazia D. Raffa, Alessandra Galati, Domenico Raimondo, Angela Rizzo, Mattia La Torre, Emanuela Micheli, Laura Ciapponi, Giovanni Cenci, Enrico Cundari, Antonio Musio, Annamaria Biroccio, Stefano Cacchione, Maurizio Gatti, Isabella Saggio

**Affiliations:** 1 Dipartimento di Biologia e Biotecnologie, Sapienza—Università di Roma, Roma, Italy; 2 Istituto Pasteur Fondazione Cenci Bolognetti, Sapienza—Università di Roma, Roma, Italy; 3 Dipartimento di Fisica, Sapienza—Università di Roma, Roma, Italy; 4 Istituto Nazionale Tumori Regina Elena, Rome, Italy; 5 Istituto di Biologia e Patologia Molecolari del CNR, Sapienza—Università di Roma, Roma, Italy; 6 Istituto di Ricerca Genetica e Biomedica del CNR, Pisa, and Istituto Toscano Tumori, Firenze, Italy; NIH, UNITED STATES

## Abstract

Telomeres are nucleoprotein complexes that protect the ends of linear chromosomes from incomplete replication, degradation and detection as DNA breaks. Mammalian telomeres are protected by shelterin, a multiprotein complex that binds the TTAGGG telomeric repeats and recruits a series of additional factors that are essential for telomere function. Although many shelterin-associated proteins have been so far identified, the inventory of shelterin-interacting factors required for telomere maintenance is still largely incomplete. Here, we characterize AKTIP/Ft1 (human AKTIP and mouse Ft1 are orthologous), a novel mammalian shelterin-bound factor identified on the basis of its homology with the *Drosophila* telomere protein Pendolino. AKTIP/Ft1 shares homology with the E2 variant ubiquitin-conjugating (UEV) enzymes and has been previously implicated in the control of apoptosis and in vesicle trafficking. RNAi-mediated depletion of AKTIP results in formation of telomere dysfunction foci (TIFs). Consistent with these results, AKTIP interacts with telomeric DNA and binds the shelterin components TRF1 and TRF2 both in vivo and in vitro. Analysis of AKTIP- depleted human primary fibroblasts showed that they are defective in PCNA recruiting and arrest in the S phase due to the activation of the intra S checkpoint. Accordingly, AKTIP physically interacts with PCNA and the RPA70 DNA replication factor. Ft1-depleted p53^-/-^ MEFs did not arrest in the S phase but displayed significant increases in multiple telomeric signals (MTS) and sister telomere associations (STAs), two hallmarks of defective telomere replication. In addition, we found an epistatic relation for MST formation between Ft1 and TRF1, which has been previously shown to be required for replication fork progression through telomeric DNA. Ch-IP experiments further suggested that in AKTIP-depleted cells undergoing the S phase, TRF1 is less tightly bound to telomeric DNA than in controls. Thus, our results collectively suggest that AKTIP/Ft1 works in concert with TRF1 to facilitate telomeric DNA replication.

## Author Summary

Chromosome ends are capped by specialized structures called telomeres, which protect chromosomes from deterioration, incomplete replication and end-to-end fusion. Defects in telomere structure and/or function may have a strong impact on human health, leading to premature aging and a variety of diseases including cancer. One of the most important tasks to understand and possibly prevent the consequences of telomere dysfunction is the identification and characterization of telomere-associated proteins. Here we show for the first time that human telomeric proteins can be identified on the basis of their homology with those that protect the telomeres of the fruit fly *Drosophila melanogaster*. Although flies and humans elongate their telomeres through different mechanisms, our studies suggested that a subset of *Drosophila* telomere-associated proteins have conserved human counterparts. Based on this hypothesis we identified and characterized a novel human telomeric protein called AKTIP. We show that AKTIP binds the components of the shelterin multiprotein complex, which caps and protects the human telomeres. AKTIP-depleted chromosomes exhibit an accumulation of DNA repair factors at their ends (telomere dysfunction foci), which are diagnostic of telomere damage. Loss of AKTIP results in a general impairment of DNA synthesis and in defective telomere replication. Collectively, our results indicate that AKTIP cooperates with the shelterin component TRF1 to ensure proper telomere replication.

## Introduction

Mammalian telomeres consist of double stranded TTAGGG repeats that terminate with a single stranded 3' overhang. These repeats are added to chromosome ends by telomerase to compensate for the terminal DNA loss that occurs at each replication cycle due to the intrinsic inability of the DNA replication machinery to duplicate chromosome ends [1]. The TTAGGG repeats bind a telomere-specific six-subunit protein complex, called shelterin, that inhibits the DNA damage response (DDR) at chromosome ends and regulates telomerase activity [2]. Three of the shelterin subunits directly interact with the TTAGGG repeats; TRF1 and TRF2 bind the TTAGGG duplex, and POT1 binds the 3’ overhang. TRF1, TRF2 and POT1 are interconnected by TIN2 and TPP1, and TRF2 interacts with hRap1, a distant homologue of *S*. *cerevisiae* Rap1 [2].

Although the shelterin components form a complex, deletions of individual shelterin subunits result in different phenotypes. For example, deletion of TRF2 activates ATM signaling and the Non Homologous End Joining (NHEJ) DNA repair pathway leading to telomeric fusions (TFs). NHEJ-induced TFs are also observed in POT1- or TPP1-depleted cells, but in this case following activation of the ATR kinase [2–5]. In contrast, loss of TRF1 activates ATR/ATM signaling and disrupts telomere replication [6, 7].

The shelterin subunits interact with several conserved polypeptides, often called shelterin accessory factors [2], which are also required for proper telomere function. These polypeptides include many proteins involved in the DNA damage response and in DNA repair such as the ATM kinase, the Ku70/80 heterodimer, the MRE11-RAD50-NBS1 (MRN) complex, Rad51, the ERCC1-XPF and MUS81 endonucleases, the Apollo exonuclease, the RecQ family members WRN and BLM, and the RTEL1 helicase. In addition, mammalian telomeres are enriched in Heterochromatin Protein 1 (HP1), the Ga9 histone methyltransferase, the Timeless component of the replisome, and the subunits of the conserved ORC and CST complexes. Losses of these shelterin accessory factors result in diverse telomere phenotypes ranging from defective telomere replication, telomere shortening, telomere loss and telomere fusion [2, 8–12].

A peculiar phenotype observed after loss of specific shelterin components or shelterin accessory factors are multiple telomeric signals (MTSs), also dubbed fragile telomeres. MTSs can be observed after fluorescent in situ hybridization (FISH) with TTAGGG probes and consist of two or more signals associated with an individual chromatid end, which normally exhibits a single compact FISH signal. MTSs have been observed after loss of several telomere-associated factors including TRF1 [6, 7] the BLM and RTEL1 helicases [6, 13], the Apollo nuclease [14–16], topoisomerase 2α (Top2α) [17], the Timeless replication factor [11], and the components of the mammalian CTC1-STN1-TEN1 (CST) complex [12, 18, 19]. Strong evidence indicates that MTSs are caused by defective telomere replication and it has been suggested that they are caused by problems in replication fork progression through telomeric DNA [6, 17–19].

We have recently identified *pendolino* (*peo*), a *Drosophila* gene that encodes an E2 variant enzyme required to prevent telomere fusion [20]. Peo interacts with terminin, a non-conserved *Drosophila* telomere-capping complex that is functionally analogous to shelterin [21–23]. Here we asked whether the human and mouse homologues (AKTIP and Ft1) of *Drosophila* Peo are required for telomere maintenance. We show that AKTIP/Ft1 is in fact needed for telomeric DNA replication and that it interacts with telomeric DNA, shelterin, and PCNA. These findings support the hypothesis [22] that “terminin accessory factors” are evolutionarily conserved proteins whose homologues might play telomere-related functions in mammals.

## Results

### AKTIP depletion affects cell cycle progression and induces the DDR and TIFs

To determine the roles of the mammalian homologues of *Drosophila* Peo (AKTIP in humans and Ft1 in mice), we produced AKTIP- and Ft1-depleted cells by lentivirus-mediated RNA interference. We generated five different hairpin sequences directed against *AKTIP* and three against *Ft1*. Infection of human primary fibroblasts (HPFs) or mouse embryonic fibroblasts (MEFs) with these recombinant lentivectors (LV-shAKTIP and LV-shFt1; henceforth abbreviated as shAKTIP and shFt1) resulted in target mRNA reductions to less than 13% of the wild type levels as measured by Q-PCR (S1A and S1B Fig). Comparable target protein reductions were observed by Western blotting in the same cells as well as in HeLa and 293T cells (S1C Fig). If not otherwise specified, in the experiments described below, we used shAKTIP11 and shFt170 infected cells (S1 Fig); uninfected cells (mock) or cells infected with a vector containing a scrambled sequence (ctr) were used as controls.

**Fig 1:**
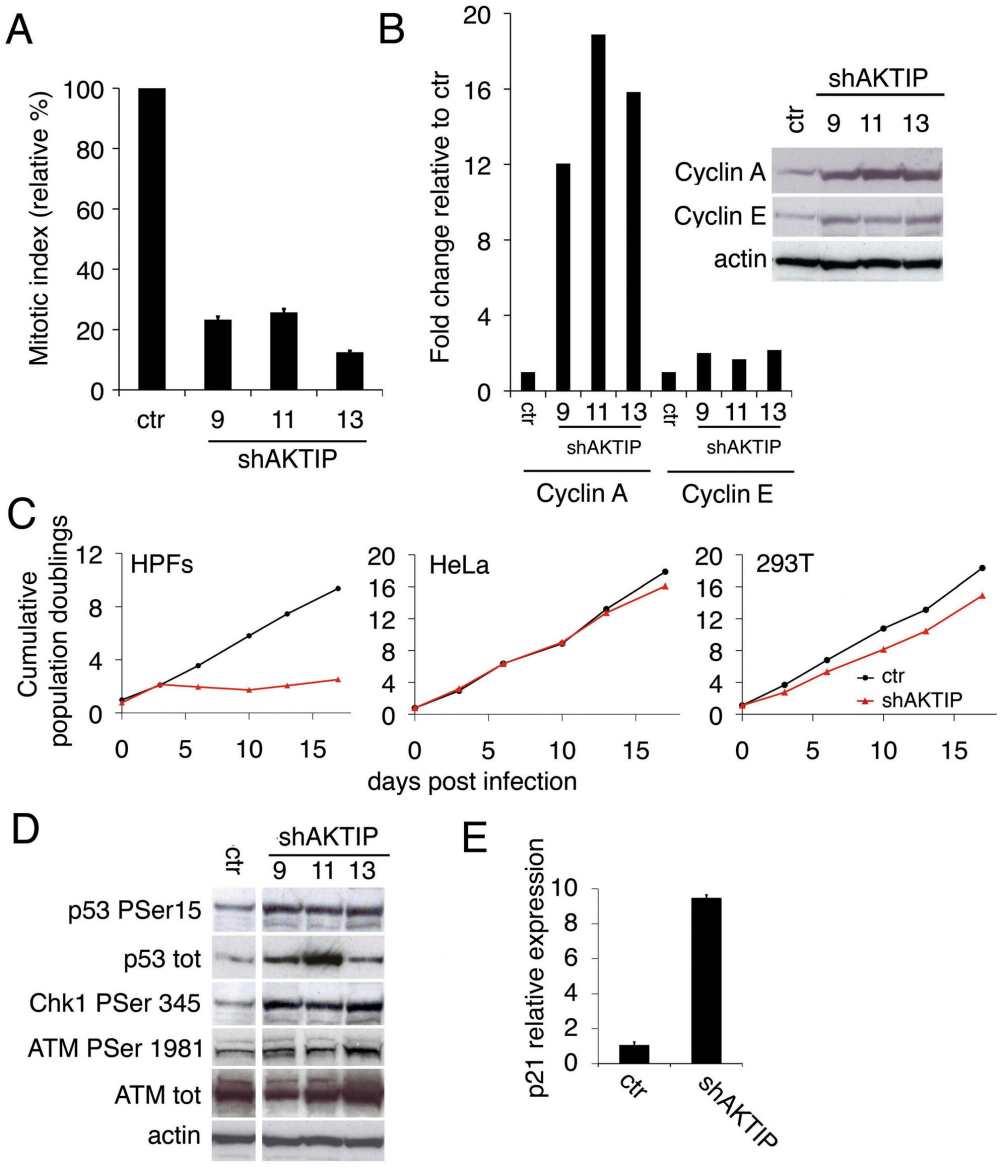
AKTIP depletion affects cell cycle progression and induces the DNA damage response (DDR). (A) Relative mitotic indexes (± SD) of shAKTIP (hairpin sequences 9, 11 and 13) and control (ctr) at 10 dpi. (B) Immunoblotting of 10 dpi extracts from shAKTIP HPFs shows accumulation of cyclins compared to ctr; in the graph, blot signals are normalized relative to the actin used as a loading control. (C) Cumulative population doublings of HPFs, HeLa and 293T cells transduced with ctr or shAKTIP-11. (D) Immunoblots of 10 dpi extracts reveal DNA damage signaling in shAKTIP samples. Densitometric analysis showed that in 9, 11 and 13 RNAi cells there is a 2.7-, 2.6-, and 3.3-fold increase of Chk1, respectively; in the same cells ATM-P Ser 1981 increases were 1.4-, 1.6- and 2.0-fold, respectively. (E) Q-PCR of total RNA shows a strong increase in p21 expression in shAKTIP-11 HPFs relative to control; samples collected at 7 dpi were analyzed in duplicate and shown as mean values ± SD. See also S1 Fig.

10 days post infection (dpi) with shAKTIP, HPFs from healthy individuals displayed a strong reduction in the mitotic index compared to controls (Fig 1A); this reduction was accompanied by a 12 to 18 fold increase in cyclin A levels, and a more modest increase in cyclin E (Fig 1B), indicating a block/delay in cell cycle progression during late S or G2 phases. Analyses of population doubling in shAKTIP-infected HPFs, 293T and HeLa cells showed that only the HPFs are strongly susceptible to AKTIP down-regulation (Fig 1C), suggesting that AKTIP depletion blocks the cell cycle by triggering p53- and pRb-dependent checkpoints, which are compromised in 293T and HeLa cells.

The AKTIP-dependent proliferation block is a likely consequence of the DNA damage response (DDR), as the phosphorylation levels of the DDR effectors ATM, Chk1 and p53 [24] were higher in shAKTIP HPFs than in controls (Fig 1D). The observation that the Chk1 phosphorylation level is substantially higher than that of ATM strongly suggests that in AKTIP-depleted cells there is a specific upregulation of the ATR/Chk1 pathway. AKTIP-depleted HPFs also showed an ~ 8 fold increase in p21 mRNA relative to controls (Fig 1E); p21 is a p53 direct transcriptional target that negatively regulates cell cycle by inhibiting cyclin-dependent kinase [25].

### AKTIP depletion induces TIFs

**Fig 2:**
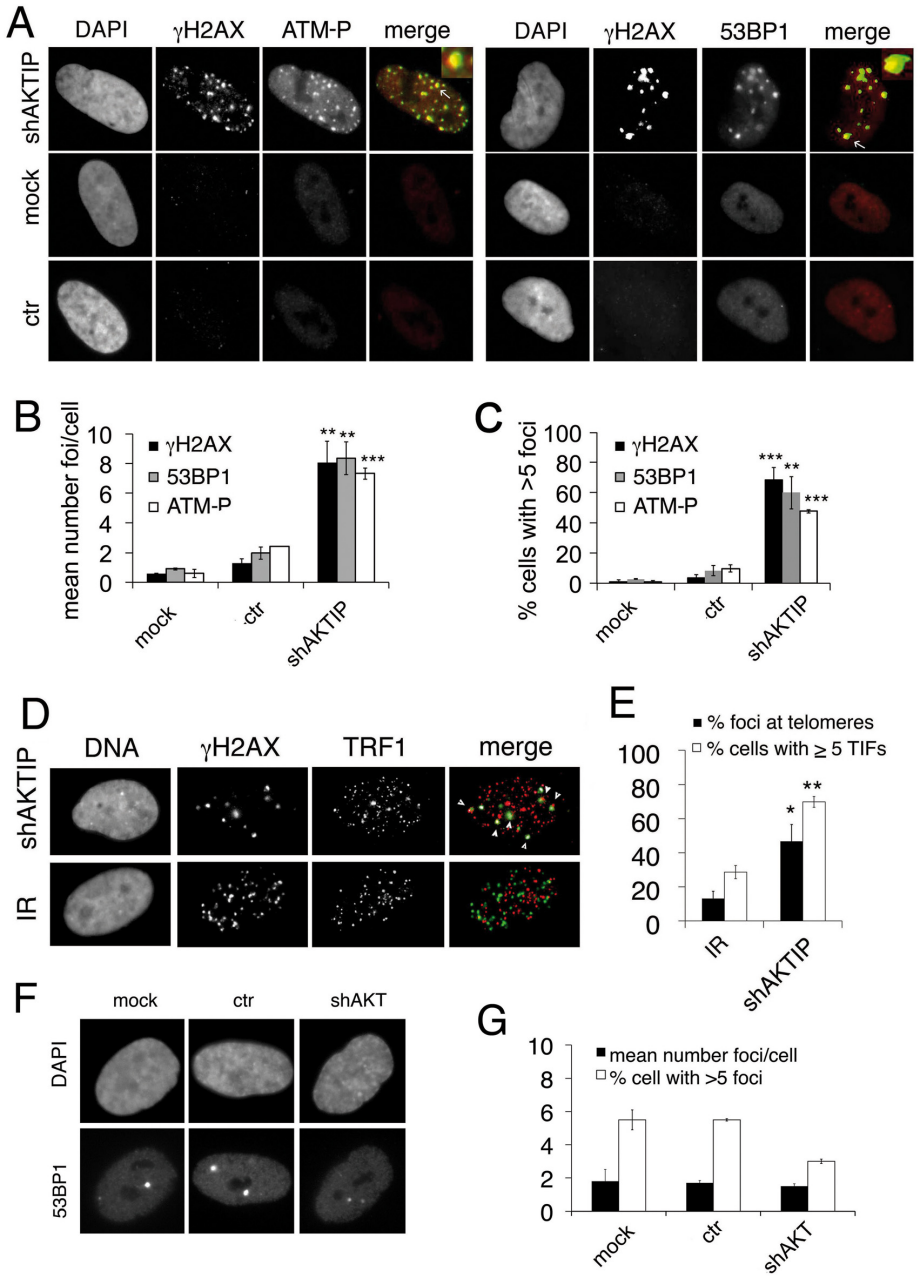
AKTIP downregulation induces TIF formation. (A) γH2AX (green in merges), ATM-P (red in merges) and 53BP1 (red in merges) foci in 5 dpi shAKTIP-11 HPFs. Foci are absent in mock and 5 dpi ctr HPFs. (B, C) Numbers of foci per cell (B) and percents of cells with more than 5 foci (C); bars are the mean values from two independent experiments ± SD. (D) TIFs in 5 dpi shAKTIP-11 HPFs (in merges, γH2AX is green and TRF1 red); arrowheads point to TIFs. X-ray-treated (IR; 1 Gy) HPFs were used as controls. (E) Percents of γH2AX foci co-localizing with TRF1 (TIFs), and of cells with more than 5 TIFs. Bars are the mean values from two independent experiments ± SD. ***, **, * indicate p<0.001 p<0.01 p<0.05 in the Student t test, respectively. See also S2 and S3 Figs. (F-G) Depletion of the AKT kinase does not induce formation of 53BP1 DNA repair foci. (F) Examples of 53BP1 foci in mock, ctr, and shAKT HPFs. (G) Quantification of 53BP1 foci in mock, ctr, and shAKT HPFs. 100 cells scored for each sample. Values are the means of two independent experiments ± SD, and are not significantly different in the Student t test.

Consistent with the DDR activation, AKTIP-depleted HPFs displayed abundant DNA repair foci containing γH2AX, 53BP1 and phosphorylated ATM (at S1981; abbreviated with ATM-P) (Fig 2A and 2B); the frequency of *AKTIP* RNAi cells with at least 5 nuclear foci was significantly higher than that observed in controls (Fig 2C). γH2AX, 53BP1 and ATM-P co-localized at most foci suggesting a coordinated response, comparable to that induced by exogenous X ray-induced DNA damage (S2 Fig).

To detremine whether the DNA repair foci seen in *AKTIP* RNAi HPFs are Telomere dysfunction-Induced Foci (TIFs, [26]), we immunostained the cells with both anti-TRF1 and anti-γH2AX antibodies. We found frequent co-localization of TRF1 and γH2AX signals (~50%), which was significantly higher than that observed in irradiated cells, where >80% of the γH2AX signals did not co-localize with telomeres (Fig 2D and 2E). TRF1 and γH2AX co-staining also verified that >60% of the AKTIP-depleted cells display more than 5TIFs/cell (Fig 2E).

Contradictory data on the existence of a biochemical link between the AKT kinase and AKTIP have been previously reported [27, 28]. We thus asked whether AKT has a role in telomere maintenance. At 10 dpi with an AKT-interfering lentivector (S3 Fig), HPFs did not display an increase in 53BP1 foci compared to controls, suggesting that AKT is not required for telomere stability (Fig 2F and 2G).

### AKTIP depletion results in fragile telomeres

**Fig 3:**
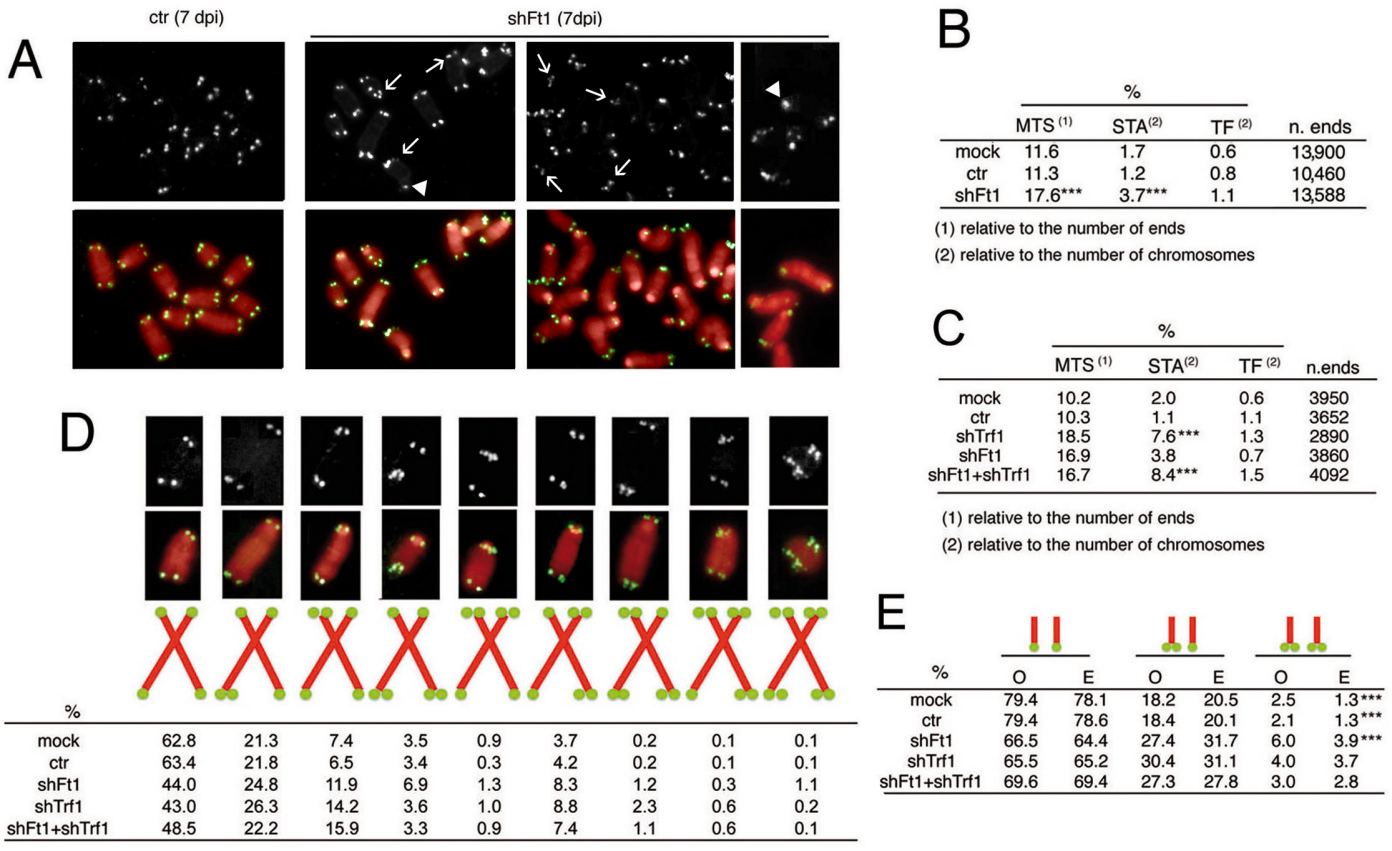
Ft1 downregulation leads to telomeric aberrations. (A) Partial DAPI-stained (red) metaphases from ctr or shFt1 MEFs showing telomeric FISH signals (black and white; green in merges); arrows indicate selected multiple telomeric signals (MTSs), and arrowhead points to sister telomere associations (STAs). (B) MTS, STA and telomere fusion (TF) frequencies in mock and 7 dpi ctr and shFt1 MEFs; *** significantly different from controls in the χ2 test with p<0.001. Values are the mean frequencies from 2 independent experiments. (C) *Trf1* is epistatic to *Ft1*. The MTS frequencies observed in shFt1, shTrf1 and shFt1 + shTrf1 are not significantly different in the χ2 test, but the STA frequencies observed in shFt1, shTrf1 and shFt1 + shTrf1 are significantly higher that that seen shFt1 (***different in the χ2 test with p<0.001). Values are the mean frequencies from 2 independent experiments. (D) Relative frequencies of the indicated chromosome types; frequencies were calculated from pooled data (B and C). (E) Observed (O) and expected (E; on the basis of independence; calculated from pooled data using the binomial distribution formula) frequencies of sister telomere pairs with the indicated FISH patterns; *** O-E differences significant with p<0.001 in the χ2 test. See also S4 and S5 Figs.

Given that *AKTIP* RNAi HPFs exhibit a very low mitotic index, to determine the telomere phenotype generated by AKTIP depletion we used p53^-/-^ MEFs (henceforth designated as MEFs), which do not undergo a cell cycle arrest following DNA damage or telomere attrition [5]. Telomeric FISH showed that 7 dpi shFt1 MEFs exhibit a significant increase in the proportion of chromatid ends with multiple telomeric signals (MTSs) with respect to controls (Fig 3A and 3B). MTSs, also dubbed fragile telomeres, have been previously observed in several settings and strong evidence exists that they are generated by defects in telomeric DNA replication [6, 15, 18, 19].

The frequency of telomeric FISH signals in *Ft1*-RNAi MEFs (95.9%) was not significantly different from those of mock (93.6%) and ctr (94.6%) controls (S4A Fig). In addition, Southern blotting analysis showed that the average length of telomeric DNA fragments from shAKTIP-transduced HPFs was comparable to that of untreated cells (S4B Fig). Thus, AKTIP depletion does not appear to cause abrupt telomere erosion. Consistent with these results, Ft1-depleted MEFs showed only a small and nonsignificant increase in TFs with respect to controls (Fig 3B). However, *Ft1* RNAi MEFs displayed a significantly higher frequency of sister telomere associations (STAs) than control cells (Fig 3A and 3B). These associations always showed a strong FISH signal at the STA site. STAs have been previously observed in telomere replication defective cells; it has been proposed that STAs are not genuine TFs generated by the DNA repair machinery but are instead the consequence of DNA bridging induced by replication stress [6, 17].

Previous studies have shown that loss of TRF1 impairs telomere replication resulting in both MTSs and STAs [6, 7]. We thus asked whether Trf1 and Ft1 function in the same pathway. The frequencies of MTSs in Trf1- or Ft1-deficient cells were comparable, and not significantly different from that observed in cells codepleted of both proteins (Fig 3C; see S5 Fig for codepletion levels). Interestingly, Trf1 deficient cells and cell co-depleted of both Trf1 and Ft1 showed an STA frequency significantly higher than that seen in cells lacking only Ft1 (Fig 3B and 3C). These results suggest an epistatic relationship between *Trf1* and *Ft1* for MTS formation. However, the relationship between these genes in STA formation is less clear. Our results only indicate that MTS and STAs arise from different forms of stress or are repaired in a different manner.

We also examined the MTS pattern in Ft1-, Trf1- and Ft1+ Trf1- depleted cells. For this analysis we pooled the data shown in Fig 3B and 3C. In control (both mock and ctr) and Ft1-depleted cells, the frequency of MTSs involving both sister telomeres was significantly higher than that expected for independent events (Fig 3D and 3E). This finding indicates that both in control and Ft1 deficient cells the leading- and lagging-strand telomeres are equally susceptible to DNA replication problems, and further suggests that at least a fraction of the MTSs observed in these cells is generated by events that simultaneously impair replication of both DNA strands. In Trf1 depleted cells, the frequency of MTSs involving both sister telomeres was not significantly different from that expected for independent events (Fig 3D and 3E). The finding that doubly depleted cells did not display a significant difference between the observed and the expected frequencies of MTSs at both sister telomeres suggests that *Trf1* is epistatic to *Ft1*, (Fig 3D and 3E). However, we cannot envisage a mechanistic explanation for the formation of an excess of fragile sister telomeres in both control and Ft1-depleted cells but not in Trf1-deficient cells.

### AKTIP interacts with telomeres

**Fig 4:**
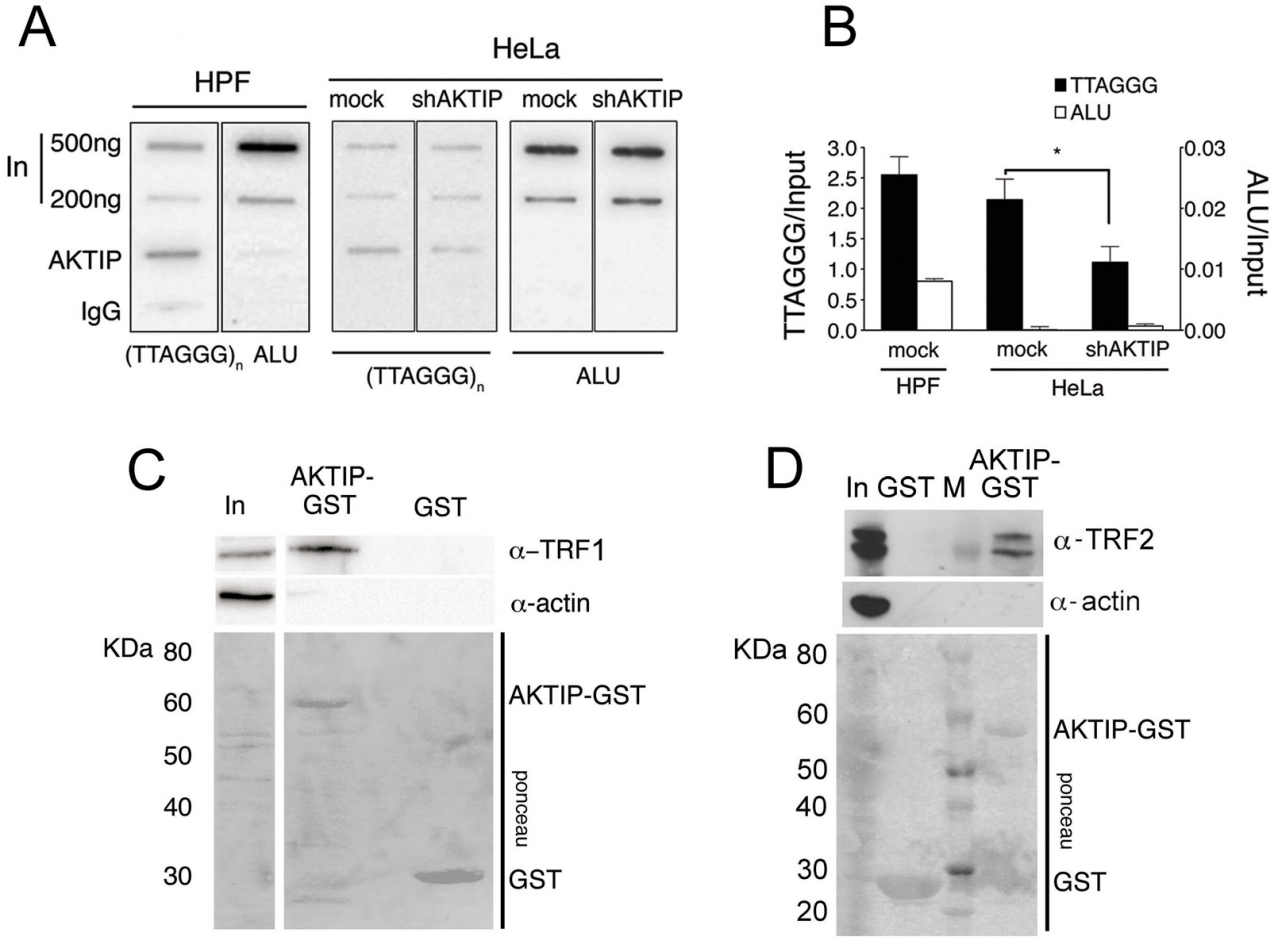
AKTIP interacts with telomeric DNA, TRF1 and TRF2. (A) ChIPs from HPFs, uninfected HeLa cells and shAKTIP-11-infected HeLa cells reveal interactions between AKTIP and telomeric DNA. Chromatin was immunoprecipitated with an anti-AKTIP antibody or control IgGs; slot-blots were hybridized with TTAGGG or ALU repeat probes. (B) ChIP quantification after normalization to the input (levels shown in A). Bars show the mean values of two experiments ± SD; the amount of telomeric DNA precipitated from uninfected HeLa cells is significantly higher than that obtained from shAKTIP-11-infected cells (*p<0.05 in the Student t test). (C, D) AKTIP-GST pulls down TRF1 (C) and TRF2 (D) from 293T cell extracts. In, input; M, MW markers.

The telomeric phenotype observed in AKTIP/Ft1 depleted cells, prompted us to investigate whether AKTIP interacts with telomeric DNA and the shelterin complex. We used chromatin IP (ChIP) to ask whether AKTIP interacts with telomeric DNA of wild type HPFs. Hybridization with a [TTAGGG]_n_ probe showed the presence of telomeric DNA in samples immunoprecipitated with an anti-AKTIP antibody (Fig 4A and 4B). A clear interaction between AKTIP and telomeric DNA was also detected in HeLa cells. The specificity of this interaction is substantiated by the absence of a detectable interaction between AKTIP and ALU DNA (in both HPFs and HeLa cells), and by the significant reduction of telomeric DNA in precipitates from AKTIP-depleted HeLa cell extracts (Fig 4A and 4B).

To determine whether AKTIP interacts with TRF1 and TRF2 we performed GST pulldown experiments from cells extracts. We found that a GST-AKTIP fusion protein precipitates both TRF1 and TRF2 from 293T cell extracts (Fig 4C and 4D).

### AKTIP directly binds TRF1 and TRF2

**Fig 5:**
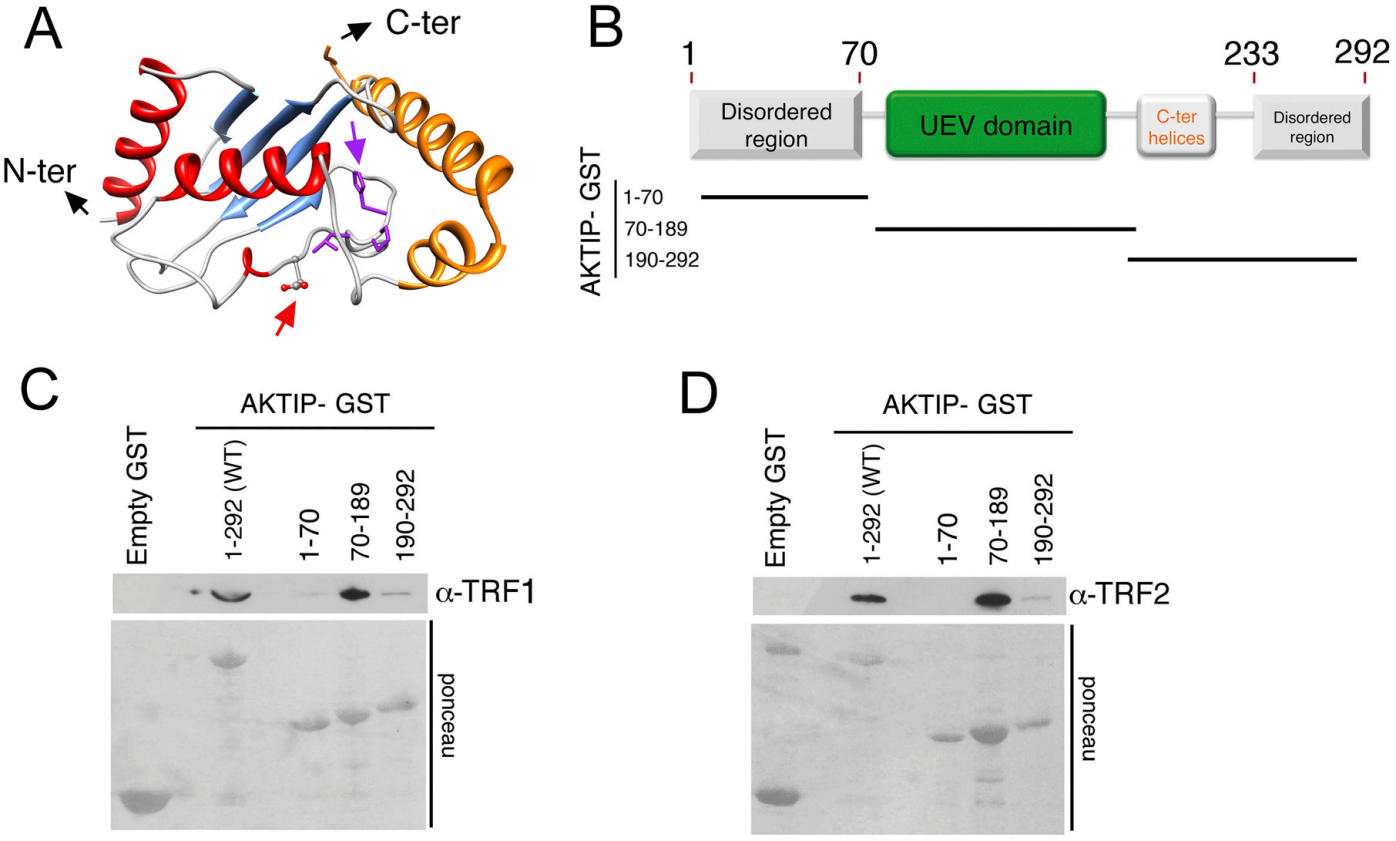
AKTIP directly binds TRF1 and TRF2. (A) A tridimensional molecular model for AKTIP. The arrows point to the starting sites of the disordered N- and C-terminal regions (not depicted); the variant Asp residue and His-Pro-Leu motif are represented as sticks and indicated by red and purple arrows, respectively (see also S5 Fig). (B) Schematic organization of the AKTIP protein; the AKTIP truncations used for GST pulldown are indicated below the scheme. (C, D) In vitro mapping the AKTIP regions that interact with TRF1 or TRF2 using bacterially purified proteins; the UEV domain of AKTIP binds both TRF1 and TRF2.

Previous studies have classified AKTIP as an E2 variant (UEV) enzyme [29]. UEVs are similar to E2 ubiquitin conjugating enzymes (UBCs) but lack the catalytic cysteine residue that is critical for the transient interaction between ubiquitin and E2. We performed a bioinformatic analysis to elaborate a three-dimensional model of AKTIP and compared this model with that of Peo, the AKTIP/Ft1 *Drosophila* orthologue required for telomere protection [20]. As shown in Fig 5A and S6 Fig, a structural comparison between the AKTIP and Peo models shows that the core UEV domains of these proteins are very similar. AKTIP and Peo, like all E2 proteins, share a canonical “core” ubiquitin-conjugating (UBC) domain of ~150 amino acids, composed of a four stranded, anti-parallel curled β-sheet. AKTIP and Peo possess additional N- and C-terminal sequences, which could be involved in regulatory functions and/or specific interactions with other macromolecules [29]. In AKTIP, the α-helices are present only on three sides of the UEV domain, while the UEV core of Peo is surrounded on four sides by α-helical segments. However, the most relevant structural difference between AKTIP and Peo is the additional N-teminal disordered region predicted only in AKTIP but lacking in Peo.

Based on the AKTIP 3D model, we constructed three protein truncations, which together define the main AKTIP structural elements (Fig 5B). GST pulldown analysis with bacterially purified proteins showed that TRF1 and TRF2 directly bind the AKTIP UEV domain but not the C-terminal helices or the disordered regions at both termini of the protein (Fig 5C and 5D and S6 Fig). These findings, together with the results of GST pulldown from cell extracts (Fig 4), indicate that AKTIP binds shelterin both in vivo and in vitro.

### AKTIP is required for general DNA replication and interacts with replisome components

**Fig 6:**
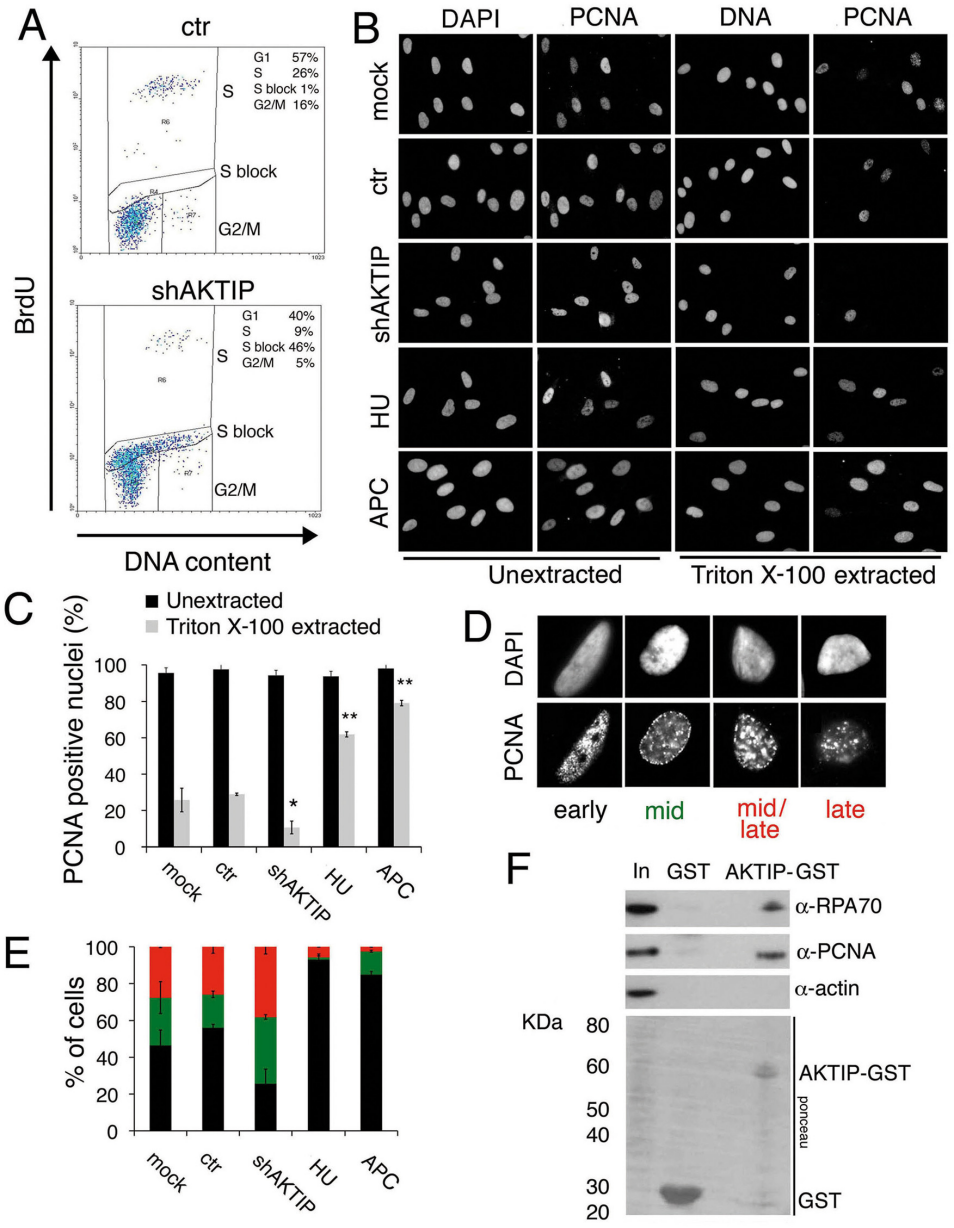
AKTIP downregulation impairs DNA replication. (A) FACS analysis of 10 dpi ctr and shAKTIP-11 HPFs incubated with BrdU for 30 min, fixed, and then stained for BrdU and DNA (with PI). AKTIP depletion results in an S phase block; percents of cells in different cell cycle phases are reported in the upper right corner of each panel. (B, C) PCNA localization in unsynchronized mock, 10 dpi ctr, 10 dpi shAKTIP-11, HU-treated and APC-treated HPFs. Examples (B) and quantification of PCNA positive nuclei (C) from unextracted or Triton X-100-extracted HPFs. Bars are the mean values ± SD of samples analyzed in duplicate. ** and * indicate significant difference from control with p<0.01 and p<0.05 in the Student t test. (D, E) Distribution of nuclei with different S phase PCNA staining patterns. Bars in the graph (E) are the mean values ± SD of samples analyzed in duplicate; colours in E are as in the representative images shown in D; distributions of ctr and shAKTIP nuclei are significantly different in the Student t test with p<0.05. (F) AKTIP-GST pulls down PCNA and RPA70 from 293T cell extracts.

The fragile telomere phenotype observed in AKTIP-depleted cells suggests that AKTIP could function in DNA replication. We thus analyzed the cell cycle distribution of unsynchronized 10 dpi shAKTIP HPFs. Flow cytometry analysis of BrdU/Propidium iodide (PI)-stained cells revealed that in shAKTIP-infected cultures 55% of the cells exhibit an S phase DNA content, 40% a G1 content and 5% a G2/M content. Only 9% of the cells showed both an S phase DNA content and BrdU incorporation, while in the remaining S phase cells (46% of the total) BrdU incorporation was not detectable. In 10 dpi control (ctr) HPFs, the S phase cells with normal BrdU incorporation were 26% of the total, the G1 cells 57% and the G2/M cells 16%; the frequency of cells with an S phase DNA content and no BrdU incorporation was only 1% (Fig 6A). These findings suggest that the cells that do not incorporate BrdU and whose DNA content is intermediate between G1 and G2 are blocked in S phase by an intra-S checkpoint triggered by DNA replication defects elicited by lack of AKTIP (see also Fig 1 above).

With these results in mind, we examined the distribution of PCNA (proliferating cell nuclear antigen) in AKTIP-depleted and control HPF nuclei. PCNA is a homotrimeric complex that encircles the DNA at the site of synthesis, acting as a processivity factor for DNA polymerases [30, 31]. As positive controls we used cells treated with hydroxyurea (HU) or aphidicolin (APC), which are known to cause cell cycle blockage in early S phase. PCNA is present in nuclei both in a soluble form that can be extracted by detergent treatment and in a detergent-resistant form (or chromatin-bound form) that is loaded onto DNA replication forks [32]. In unextracted nuclei, nearly 100% of control, AKTIP-depleted, HU-treated or APC-treated nuclei showed a PCNA signal (Fig 6B and 6C). In contrast, after Triton X-100 extraction, 30% of control nuclei, 9% of AKTIP-depleted nuclei, 64% of HU-treated nuclei and 80% of APC-treated nuclei displayed PCNA staining. These results indicate, as expected, that PCNA associates with replication forks in S phase control nuclei and in the nuclei of cells arrested in early S following either HU or APC treatment. More importantly, our results strongly suggest that the AKTIP-depleted HPFs that exhibit an S phase DNA content but fail to incorporate BrdU (Fig 6A) do not contain chromatin-bound PCNA.

We also analyzed PCNA localization in detergent-extracted nuclei. Previous studies have shown that PCNA marks DNA replication foci that change their position during the S phase. Early S is characterized by numerous small PCNA foci concentrated in the inner part of the nucleus; in mid-S the PCNA foci decrease in number and move toward the nuclear periphery; and in late S, the foci increase in size and become scattered throughout the nucleus [32, 33]. In control HPFs, we observed distributions of PCNA foci that are fully consistent with published results [33] and allow subdivision of the S phase nuclei into four categories (early-S, mid-S, mid/late-S, and late-S; Fig 6D). Examination of PCNA foci in detergent-extracted nuclei revealed that AKTIP-depleted cells display significantly higher proportions of nuclei in mid- and mid/late-S than controls (Fig 6E). As expected, most of the nuclei of HU- or APC-treated cells were found to be in early S phase. These results indicate that AKTIP-depleted S phase cells, even if they still incorporate BrdU, tend to be delayed in their progression through the S phase and thus accumulate in mid- and mid/late-S.

We finally asked whether AKTIP physically interacts with PCNA and RPA70, which is another well-known component of the DNA replication machinery. GST pull down experiments using AKTIP-GST and 293T cell extracts revealed that AKTIP precipitates both PCNA and RPA70 (Fig 6F). It should be noted that our bioinformatic analyses did not detect PIP or APIM motifs in the AKTIP protein. These motifs are known to mediate contacts between PCNA and its interacting proteins but there is also evidence that PCNA can contact partner proteins independently of these motifs (reviewed [31]). Regardless the precise nature of the AKTIP-PCNA interaction, our data collectively indicate that AKTIP is required for DNA synthesis, and strongly suggest that in the absence of AKTIP human primary fibroblasts arrest in interphase due to the activation of the S phase checkpoint.

### AKTIP is required for efficient telomere replication and functions in concert with TRF1

**Fig 7:**
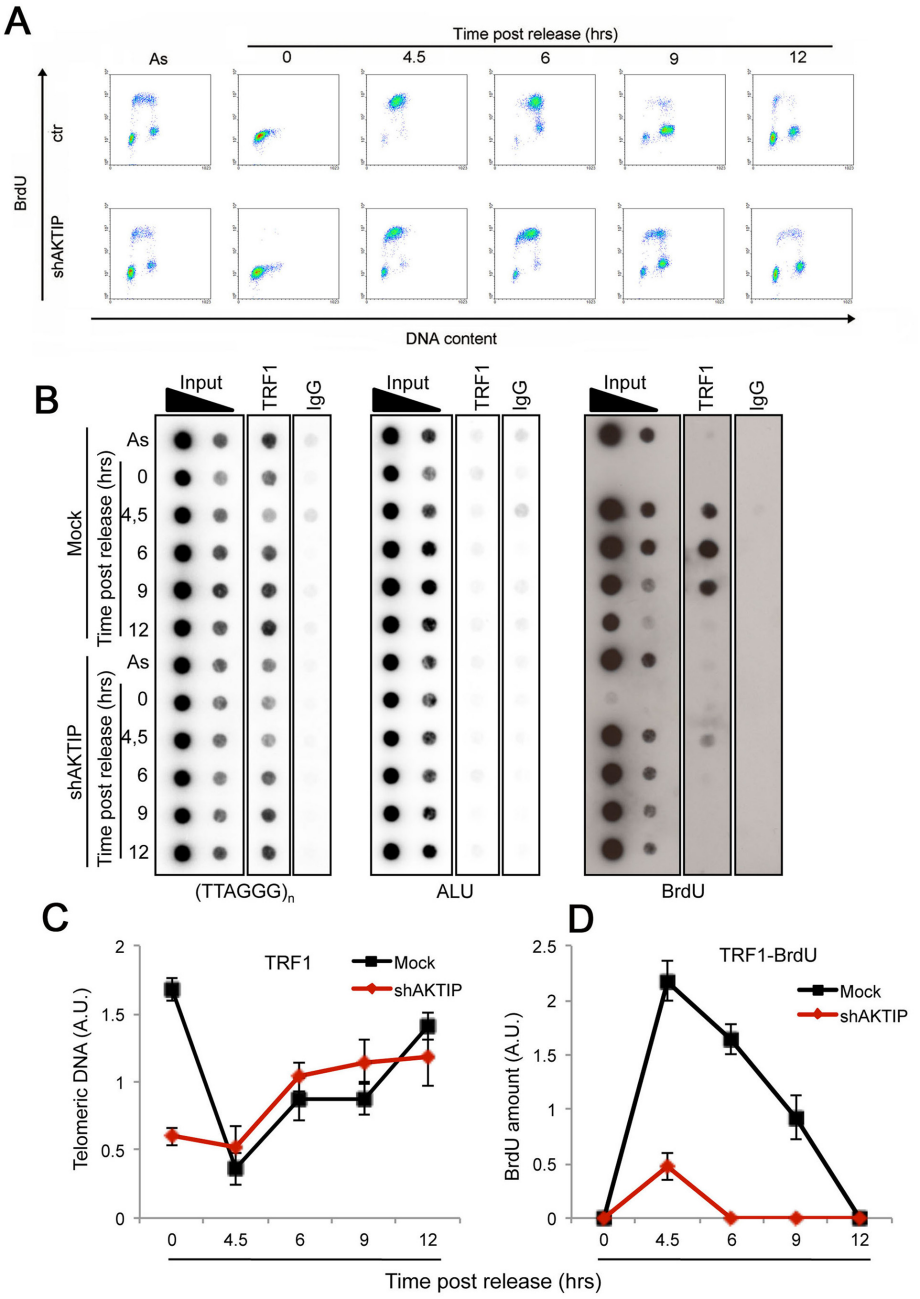
AKTIP downregulation impairs telomere replication. Ctr or shAKTIP HeLa cells were synchronized at the G1/S boundary with a double thymidine block and harvested at the indicated times. (A) Scatter plots showing the proportions of cells in S phase in asynchronous cultures (As) and in cultures analyzed at various times after release from the double thymidine block. Prior to harvest at each time point, the cells were incubated with BrdU for 30 min. (B) ChIP analysis on synchronized HeLa cells incubated with BrdU for 1 h before harvesting. Precipitations were performed with an anti-TRF1 antibody. IgG antibody was used as negative control. Inputs represent 10 and 1% of genomic DNA. Dot-blot analysis was performed using telomeric or ALU repeat-specific probes. Precipitated DNA was analyzed by Western blotting with an anti-BrdU antibody. (C, D) Quantification of the data expressed in arbitrary units (A.U.) of unlabeled (C) or BrdU-labeled (D) precipitated telomeric DNA at the different time points of analysis, each normalized to input samples. The graphs show three independent experiments, with error bars indicating the SD.

Altogether, the findings that loss of AKTIP/Ft1 arrests cells in the S phase and results in fragile telomeres, and that AKTIP interacts with PCNA and RPA70 suggest that AKTIP/Ft1 is involved in telomere replication. In addition, our results indicated an epistatic relationship between AKTIP and TRF1, which is also required for telomere replication [6, 7]. To address the relationships between AKTIP and TRF1 during telomere replication we combined BrdU incorporation (to mark replicating DNA) and chromatin immunoprecipitation (ChIP) with anti-TRF1 antibodies. Hela cells were synchronized with a double thymidine block and harvested at various times after release from the G1/S block; before harvest the cells were incubated with BrdU for 1hr. The proportions of cells in S-phase at each post-release time were determined by FACS analysis based on BrdU incorporation (Fig 7A). An examination of the scatter plots indicates that the overall DNA synthesis is delayed in AKTIP-depleted cells compared to mock controls. In addition, as shown in Fig 7B (right panel) and 7D, the chromatin fragments immunoprecipitated by TRF1 at the 4.5, 6 and 9 hrs post-release times contain almost no BrdU compared to controls. Because AKTIP-depleted HeLa cells do not exhibit gross proliferation defects (Fig 1C), this finding cannot be interpreted as indicating a complete failure of telomere replication, as this event would cause profound defects in telomere structure leading to cell cycle arrest. Thus, the most likely interpretation is that in AKTIP-depleted cells undergoing the S phase TRF1 is less tightly bound to telomeric DNA than in controls. This interpretation is corroborated by the observation that in AKTIP-depleted cells arrested in early S by the double thymidine block there is less telomere-bound TRF1 than in controls (Fig 7B and 7C). However, at the 4.5, 6 and 9 hrs post-release times AKTIP-depleted and control cells yielded similar amounts of TTAGGG precipitates (Fig 7B and 7C). This latter result is not conflicting with our interpretation as only a small fraction of telomeres is expected to undergo replication at the time unit sampled in the experiment [34]. In addition, at least a fraction of the TTAGGG precipitates obtained from AKTIP-depleted cells at the 4.5, 6 and 9 hrs post-release times might be a consequence of replication-independent loading of TRF1 on partially/aberrantly replicated telomeres [35]. Collectively, these results suggest the hypothesis that AKTIP is required for proper TRF1 association with telomeres during their replication.

### AKTIP is enriched at the nuclear periphery

**Fig 8:**
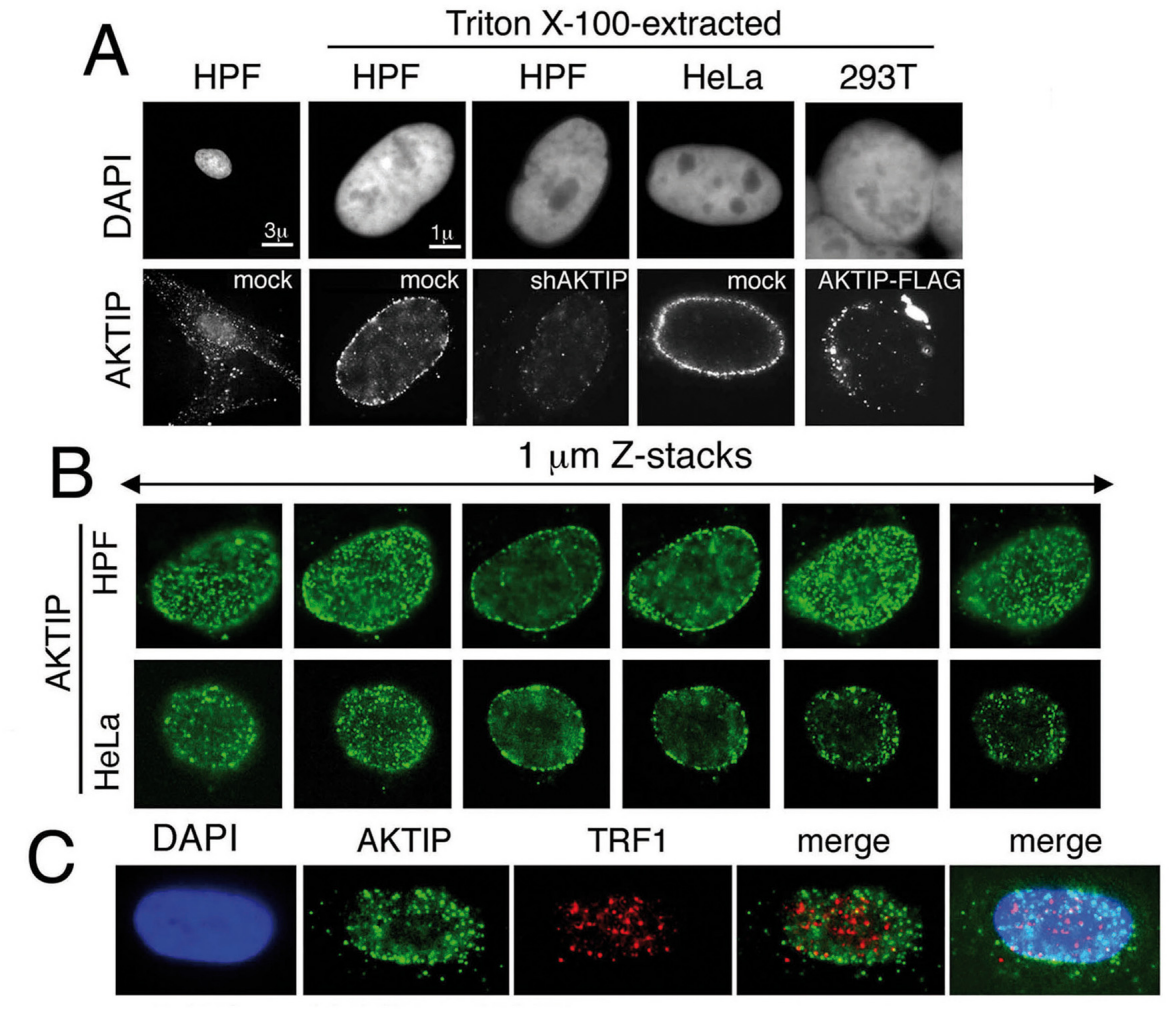
AKTIP localizes at the nuclear periphery. (A) Immunolocalization of AKTIP in HPFs and HeLa cells with an anti-AKTIP antibody, and in AKTIP-FLAG expressing 293T cells with an anti-FLAG antibody. shAKTIP HPFs show a strong reduction of the AKTIP signal. (B) Optical sections of a HPF and a HeLa cell showing AKTIP enrichment at the nuclear periphery. (C) Co-immunostaining of detergent-extracted HPFs for AKTIP and TRF1 (projection of 8 z stacks) showing a limited signal co-localization.

To obtain further insight into the role of AKTIP at telomeres, we examined its subcellular distribution. Human cells were immunostained with an anti-AKTIP antibody using standard methods or after protein extraction with Triton X-100, a procedure used to detect subnuclear localization of proteins [36]. In unextracted cells, AKTIP was found in both the nucleus and the cytoplasm. In detergent-extracted cells, including HPFs, HeLa and 293T cells, AKTIP was only nuclear and was enriched near the nuclear rim in a punctate pattern (Fig 8A and 8B). The AKTIP signal was strongly decreased in shAKTIP cells, confirming the specificity of the anti-AKTIP antibody (Fig 8A). Preferential AKTIP localization at the nuclear periphery was also detected in 293T cells transfected with an AKTIP-FLAG-expressing vector and stained with an anti-FLAG antibody (Fig 8A). Examination of optical sections from 50 randomly chosen detergent-extracted cells immunostained for AKTIP did not reveal substantial differences in AKTIP distribution within the nucleus; AKTIP was consistently enriched at the nuclear periphery in all cells. This observation suggests that AKTIP does not undergo gross variations in its subnuclear localization during the cell cycle.

We next asked whether AKTIP colocalizes with telomeres. Examination of 23 asynchronously growing HPF nuclei stained for both AKTIP and TRF1 (Fig 8C) revealed that the frequency of co-localization of TRF1 and AKTIP signals ranges from 5 to 25%. These results are consistent with a transient telomere-AKTIP interaction during the S phase.

## Discussion

AKTIP is a UEV protein that contains a UBC domain lacking the catalytic cysteine that mediates ubiquitin transfer. Studies on UEV proteins have shown that in many cases their UEV domains can bind ubiquitin and play roles in the ubiquitylation-related processes [37]. However, UEV proteins serve also functions that that do not appear to be mediated by their UEV domains and that are likely to be unrelated to the ubiquitin pathway. For example, a series studies on TSG101 (the product of *Tumor susceptibility gene 101*) has implicated this UEV protein in diverse cellular functions including cell cycle regulation, endosomal trafficking, transcription, cytokinesis, and viral budding (reviewed in [38]). Similarly, two studies on AKTIP/Ft1 have suggested that this protein promotes AKT activity as pro-apoptotic factor [27] and mediates vesicle trafficking and/or fusion [28].

We have shown that AKTIP is required to prevent spontaneous DNA damage and TIF formation. AKT depletion does not cause DNA repair foci indicating that this kinase is not involved in telomere protection. However, this result does not exclude roles of AKTIP in AKT regulation and vesicle trafficking. We have further shown that AKTIP interacts with telomeric DNA and binds the shelterin components TRF1 and TRF2 both in vivo and in vitro. HPFs with strongly diminished AKTIP levels displayed a strong proliferation defect and arrested in late S phase showing a substantial reduction in chromatin-bound PCNA. Consistent with these results, we found that AKTIP physically interacts with both PCNA and RPA70. No substantial growth defects were observed in immortalized MEFs or human cancer lines treated with *Ft1/AKTIP*-interfering lentivectors. However, compared to controls, MEFs showed a strong increase in MTSs, and a more modest increase in STAs, suggesting a defect in telomere replication. Collectively, these results indicate that *AKTIP/Ft1*, the mammalian hortologue of *Drosophila peo* [20], plays a conserved function required for DNA replication and for telomere maintenance.

### Loss of AKTIP/Ft1 results in fragile telomeres

TIFs and impaired cell proliferation are common phenotypes seen after loss of shelterin components or shelterin accessory factors. In contrast, MTSs—or fragile telomeres- have been only observed after loss of specific telomere factors including TRF1, the BLM and RTEL1 helicases, the Apollo nuclease, topoisomerase 2α (Top2α), the replisome-associated Timeless protein, and the components of the mammalian CTC1-STN1-TEN1 (CST) complex (see introduction). Strong evidence indicates that MTSs are caused by defective telomere replication [6, 17–19], and single DNA molecule analysis has shown that in the absence of TRF1 replication forks tend to stall when they encounter telomeric DNA [6].

Deficiency of proteins involved in telomere replication leads to different telomere-related phenotypes. Depletion of either TRF1 or BLM results in frequent MTSs but not in telomere loss; in contrast, lack of Apollo, CST complex or RTEL1 produces both MTSs and telomere loss [6, 9, 12–16, 19]. In CTC1- or RTEL1-depleted cells, telomere loss is probably due to the formation of telomeric DNA circles resulting from the excision of the t-loop [13, 18]. It has been thus proposed that factors like RTEL1 perform two distinct functions: they favor t-loop disassembly and help unwind G4-DNA structures during telomere replication [13]. TRF1, BLM and the CST component TEN1 do not appear to prevent t-loop excision [12, 13].

The main telomere aberrations produced by loss of AKTIP/Ft1 are MTSs and STAs; we did not observe a significant increase in either telomere loss or telomere fusion. Thus, the phenotype elicited by loss of AKTIP/Ft1 is very similar to the phenotype observed in TRF1- or BLM-deficient cells. We have also shown that cells co-depleted of Trf1 and Ft1 exhibit an MTS frequency comparable to that observed in cells depleted of either Trf1 or Ft1 only, suggesting that both factors function in the same telomere replication pathway. In addition, previous analysis of cells simultaneously deficient of both TRF1 and BLM revealed an epistatic relationship in the MTS formation pathway [6]. In contrast, TRF1 and the CST complex appear to function in different pathways; co-depletion of TRF1 and STN1 resulted in greater than additive increase in the MTS frequency relative to those observed in cells depleted of either of these proteins [19]. Thus, our results suggest that AKTIP/Ft1 works in concert with TRF1 to facilitate telomeric DNA replication, while it is not required to prevent t-loop excision.

### The role of AKTIP/Ft1 in DNA and telomere replication

Consistent with the cytological data, we showed that AKTIP/Ft1 is required for genome wide and telomere replication. However, immunostaining of nuclei of asynchronous HPFs revealed that the frequency of TRF1 spots that co-localize with AKTIP signals ranges from 5 to 25%. We believe that this low colocalization frequency is in line with our Ch-IP results (Fig 7), which point to an increased association of AKTIP with telomeres during their replication. Previous studies have shown that human telomeres replicate throughout the S-phase with telomere-specific time windows, and that individual telomeres can replicate in less than one hour [34]. Thus, given that the S phase lasts about 6 hours, if AKTIP associated with telomeres only during their replication, the observed TRF1/AKTIP colocalization frequency would be compatible with the expected one. In summary, our results indicate that Ft1/AKTIP plays a genome-wide role in DNA replication and is an important component of the molecular machinery that facilitates mammalian telomere replication.

Our results suggest a simple model for the role of AKTIP/Ft1 in telomere replication. It has been proposed that TRF1 recruits/activates the BLM and RTEL1 helicases that help unwind G4 DNA structures during TTAGGG repeat replication [6, 13]. Our results suggest the hypothesis that the AKTIP-TRF1 interaction helps TRF1 to maintain a tight association with telomeric DNA during its replication. It thus conceivable that AKTIP/Ft1 depletion impairs the interaction of TRF1 and its associated helicases with the telomeric G4 structures compromising the replication process.

### Evolutionary conservation of the AKTIP/Ft1 function

Mammalian telomere proteins have been isolated through the analysis of biochemical interactions between different telomere components, or on the basis of their homology with telomeric proteins identified in organisms with telomerase such as yeasts. AKTIP/Ft1 is the first mammalian telomere factor isolated because of its homology with a telomeric protein identified in *Drosophila*, an organism without telomerase in which telomeres are elongated by a transposition-based mechanism. The rationale for using *Drosophila* as model system to detect new mammalian telomere factors was suggested by recent studies on the organization and evolution of fly telomeres. *Drosophila* telomeres are capped by the non-conserved, fast evolving and telomere-specific terminin complex, which appears to be functionally analogous to shelterin [21–23, 39]. *Drosophila* telomeres are also protected by a number of conserved “terminin accessory” factors, which include HP1a, ATM, Mre11, Rad50, Nbs, the E2 enzyme UbcD1, the Woc transcription factor and Peo [20, 22]. We proposed previously that concomitant with telomerase loss *Drosophila* rapidly evolved terminin to bind chromosome ends in a sequence-independent fashion, and that terminin accessory factors did not evolve as rapidly as terminin because of the functional constraints imposed by their involvement in diverse cellular processes [22]. This hypothesis suggests that terminin accessory factors might correspond to ancestral telomere-associated proteins with homologues in other organisms including mammals. Our results on AKTIP/Ft1 provide a strong support for this idea, showing that the human homologue of the non-terminin protein Peo is required for telomere maintenance. Strikingly, Peo and AKTIP directly bind terminin and shelterin, respectively, although the proteins that comprise these complexes do not share any homology. This finding highlights the importance of Peo/AKTIP/Ft1 as telomere maintenance factor, as the protein co-evolved with divergent capping complexes to maintain a direct interaction with telomeres.

Although our hypothesis on *Drosophila* telomere evolution posits that terminin accessory factors play conserved telomere-related functions, our results indicate that Peo and AKTIP/Ft1 play similar but non-identical roles in telomere maintenance. Both Peo and AKTIP/Ft1 are required for DNA replication and for stable PCNA binding to replicating chromatin. However, while Peo is required to prevent telomere fusion AKTIP/Ft1 does not appear to serve a similar function. Previous studies identified several other factors (HP1, ATM, Rad50, Mre11 and Nbs) with major roles in preventing telomere fusion in flies but not in humans (reviewed by [8, 22, 40]). These findings are intriguing and suggest that TF prevention in flies requires more factors than those that are normally required to avoid mammalian telomere fusion. A possible explanation for this requirement is that the sequence independent *Drosophila* telomeres, which are unlikely to form a protective telomere loop, need a more complex capping machinery than their mammalian counterparts. Regardless of the correctness of this hypothesis, our results on AKTIP/Ft1 suggest that the identification of additional terminin accessory factors might lead to the discovery of novel human telomere components.

We have shown that mutations in *peo* cause TFs that preferentially involve the telomeres associated with constitutive heterochromatin, providing the first demonstration that subtelomeres can affect telomere fusion [20]. Studies on mammalian cells have shown that the subtelomeric regions affect the telomere replication time [34, 41] but never addressed whether the fusigenic properties of different chromosome ends depend on subtelomers. Here, we could not ask this question because AKTIP/Ft1 depletion does not results in TFs. However, we believe that investigating the role of mammalian subtelomeres in telomere fusion is and interesting research topic that should be addressed by future studies.

## Materials and Methods

### Cell culture and LV-mediated RNAi

Human foreskin primary fibroblasts (HPFs), p53^-/-^ MEFs [42], HeLa (ATCC CCL-2) and 293T (ATCC CRL-11268) cells were cultured in DMEM with 10% FBS (Invitrogen). Lentivirus (LV) production and infection were performed as in [43]; RNAi was carried out using a shRNA vector (S1 Table), and the pCMV-dR8.74 and pMD2.G vectors (http://www.addgene.org). For all viruses the transfer vector backbone was PLKO.1 (Sigma). The LV-mediated RNAi efficiency in all cell types used here remained unchanged for many days post-infection. In the course of the experiments we consistently performed Western blotting and/or Q-PCR to assess the level of AKTIP/Ft1 in RNAi cells. We did not observe substantial variations in these levels in a 27-day period, starting from 24 hours post-infection.

Population doubling (pd) was calculated with the formula Log(n_t_/n_0_)x3.33, where n_0_ is the number of cells plated and n_t_ the number of cells at the n dpi. Cells were irradiated with 1Gy (0.28 Gy/min.) of X-rays. Where indicated, cells were treated for 18 h with 2mM hydroxyurea (Sigma) or 24 h with 1μM aphidicolin (Sigma). AKTIP-FLAG-expressing 293T cells were obtained by transfection of the pCMV6-Entry-AKTIP-FLAG plasmid (OriGene).

### Cell synchronization

Cells were synchronized at the G1/S boundary using a double-thymidine block. Cells were treated with 2 mM thymidine for 14 h, and then released to fresh medium for 10 h followed by second treatment with 2 mM thymidine for 14 h. In AKTIP-depleted cells, the synchronization protocol started at 1 dpi.

### FACS analysis

Cells incubated for 30 min in 45 μM BrdU were fixed in 70% cold ethanol for 30 min., washed in PBS/0.5% Tween 20 and treated with 3M HCl for 45min. Cells were then stained with the anti-BrdU monoclonal antibody (Dako) and a secondary Alexa-Fluor488-conjugated antibody (Jackson), and counterstained with Propidium Iodide (PI, Sigma) 20μg/ml. Acquisition was carried out using a Beckman-Coulter Epics XL flow-cytometer; data were analyzed by the WinMDI software.

### Immunostaining, FISH and cytology

For immunostaining, cells were fixed with 3.7% formaldehyde for 10 min at 4°C and permeabilized with 0.25% Triton X-100 in PBS for 5 min. Where indicated, cells were pre-permeabilized according to [26]. Cells were then incubated with the following antibodies in the presence of 3% BSA: anti-ATM-pS1981 (Rockland Immunochemicals), anti-53BP1 (Novus Biologicals), anti-γH2AX (Upstate Biotechnology), anti-AKTIP (Sigma), anti-FLAG (Sigma), anti-TRF1 (a gift of T. de Lange, Rockefeller University NY), or anti-PCNA (Santa Cruz). Primary antibodies were detected by 45 min incubation at RT with the following secondary antibodies: anti-mouse-FITC (Jackson Immunoresearch), anti-mouse-Rhodamine (Jackson Immunoresearch), anti-rabbit-ALEXA 555 (Invitrogen) or anti-goat-FITC (Jackson Immunoresearch).

Mitotic index was calculated by examination of HPFs incubated for 3 h with colchicine (Sigma), treated with KCl 75mM for 7min and fixed with methanol: acetic acid 3:1 for 15 min. Preparations for mitotic index analysis and immunostained preparations were mounted in DAPI-Vectashield (Vector laboratories) to stain DNA and chromosomes.

FISH was carried out according to [44], and the telomeric probe was obtained by PCR as described by [45]. PCR products were then sonicated to obtain 500–2000 bp fragments. After the hybridization reaction, the slides were washed 3 times in SSC 4X- 0.1% TWEEN-20, air-dried and then mounted in DAPI-Vectashield.

FISH was examined with a Zeiss Axioplan epifluorescence microscope equipped with a CCD camera (CoolSnap HQ; Photometrics,). TIFs were detected using a spinning-disk confocal (CarvII, Beckton Dickinson) microscope. Fluorescent optical sections, captured at 1μm Z steps using the same spinning-disk microscope, were examined separately or as a maximum-intensity projection.

### Q-PCR

Cells were lysed at 7 dpi using the TRIzol reagent (Invitrogen); RNA was prepared according to the manufacturer's instructions and reverse transcribed using an oligo d(T) primer and the OMNISCRIPT RT KIT (Qiagen). Target gene expression was quantified according to [43] using specific primers (S2 Table) selected with the Primer Express software (Applied Biosystems).

### Southern blotting

For each sample, 3μg of DNA extracted from HPFs with Nucleospin Tissue Genomic DNA isolation kit (Clontech) were cleaved with Hinf I/Rsa I (Roche) and separated in a 0.7% agarose gel. Fractionated DNA was depurinated by treatment with HCl 0.25 M for 20 min, denaturated with NaCl 1.5M-NaOH 0.5M for 40 min, and neutralized in NaCl 1.5M-TrisHCl 0.5M (ph 7.5) for 40 min. DNA was then transferred to Nytran-N membrane (Whatman) in 20x SSC by overnight incubation. The membrane was backed at 80°C for 2 h. Hybridization was carried out overnight at 47.8°C using a TTAGGG repeat probe obtained according to [45]; membranes were then washed with 2X SSC -0.1% SDS at RT and then with 0.2X SSC -0.1% SDS at 50°C. Telomeric DNA was visualized using the DIG Luminescent detection Kit (Roche) according to the manufacturer’s instructions.

### Immunoblotting

Samples were treated with lysis buffer [Tris–HCl 50mM pH7.4, 10% NP-40, 0.25% NaDesoxycholate, EDTA 1mM, NaCl 150mM, PMSF 1mM, protease inhibitor cocktail (Roche)] and loaded onto pre-cast 4–12% gradient acrylamide gels (NuPAGE, Invitrogen). After electro-blotting, filters were incubated with anti-AKTIP (Sigma), anti-TRF2 (Novus Biologicals), anti-actin-HRP conjugated (Santa Cruz), anti-cyclin A (Santa Cruz), anti-cyclin B (Santa Cruz), anti-cyclin E (Upstate Biotechnology), anti-p53-pSer15 (Cell Signaling Technology), anti-p53 (DakoCytomation), anti-ATM-pS1981 (Rockland Immunochemicals), anti-ATM (Genetex), anti-ChK1-PSer345 (Cell Signaling Technology), anti-AKT (Cell Signaling Technology), anti-PCNA (Santa Cruz), anti-RPA70 (Santa Cruz), or anti-TRF1 (Santa Cruz). Filters were then incubated with appropriate HRP-conjugated secondary antibodies (Santa Cruz), which were detected using the enhanced chemiluminescence system (ECL plus, Amersham). Signals were quantified with Image J software.

### GST-pulldown

Full length *AKTIP* and *AKTIP* fragments (Fig 5), were amplified by PCR using the primers listed in S3 Table and cloned in the pGEX6p1 vector (GE Healthcare) for expression in bacteria. Bacterially expressed GST fusion proteins were purified using QIAGEN Glutathione HiCap Matrix according to the manufacturer’s instructions. GST pulldown from 293T cell extracts was carried out as previously described [21]. For the analysis of direct interactions between bacterially expressed proteins, AKTIP-GST recombinant polypeptides were incubated in NETN buffer (20 mM Tris-HCl, pH 8, 100 mM NaCl, 1 mM EDTA, 0.5% N P-40) with either TRF1 or TRF2, produced and purified as previously described [46]. Complexes were collected by centrifugation, washed 3 times with NETN buffer, and electroblotted as described above. TRF1 and TRF2 were detected with anti-TRF1 (Santa Cruz) and anti-TRF2 (Imgenex) antibodies.

### ChIP

Cross-linking was carried out by treating HPFs or HeLa cells with 1% formaldehyde for 15 min; the reaction was stopped with 0.125 M glycine. Cells were then lysed, and chromatin was extracted according to Galati et al. [47]. Chromatin was then incubated overnight with 7.5 μg of mouse monoclonal anti-AKTIP (Sigma), 1μg of mouse IgG (Sigma), 7μg anti-TRF1 antibody (Santa Cruz), or 1μg of goat IgG (Santa Cruz) at 4°C, and ChIP was carried out as described [47]. DNA was slot-blotted onto a Hybond N+ and hybridized with a 650 bp telomeric probe from a plasmid containing a 1.6 Kb of TTAGGG repeats (a gift of E. Gilson), or with an ALU probe obtained by genomic DNA amplification with the 5’-CGCCTGTAATCCCAGCACTTTG-3’ and 5’-ACGCCATTCTCCTGCCTCAGC-3’ oligos. Signals were quantified using the ImageQuant Software.

For the BrdU-ChIP assay, before cell harvesting at each time point, the cells were incubated with 20 μM BrdU (Sigma) for 1 h. After dot-blotting and before hybridization with the telomeric probe, BrdU incorporation into telomeric DNA was evaluated by western blot analysis by incubating the membrane with the primary anti-BrdU antibody (Becton Dickinson).

### Bioinformatic analysis of the AKTIP structure

The AKTIP tridimensional model was elaborated following the same procedure used for the construction of Peo model [20]. Briefly, we used CSI-BLAST and the CLUSTALW software to obtain a multiple sequence alignment (MSA), which served to construct a Hidden Markov Model (HMM) of the protein family. Searching the Pfam database with this HMM yielded the UBC/E2 enzyme family; the second hit was the UEV family that includes Tsg101, Mms2, UEV1and Peo. AKTIP contains an aspartic acid residue (at position 106, according to SwissProt numbering) in place of the E2 catalytic cysteine. In addition, 8 residues before the site of catalytic cysteine site, AKTIP exhibits an HPL tripeptide (HPH in Peo) instead of the HPN peptide, which is a canonical signature of the E2 superfamily. Prediction of potentially disordered regions using the GeneSilico MetaDisorder server revealed that at the N and C termini of AKTIP there are stretches of ~ 70 aa that have a tendency to be intrinsically disordered. AKTIP modeling was performed using the composite approach implemented in I-TASSER server [48] and refined using the HAAD software FG-MD algorithm [49, 50]. The AKTIP model was evaluated a potentially extremely good model (with a predicted LGscore of 5.9) by the PRO-Q model quality assessment program [51], and its QMEAN score [52] was 0.8 (the variability range is 0–1, with 1 being a perfect model).

## Supporting Information

S1 Fig RNAi-mediated AKTIP and Ft1 downregulation. (A, B) *AKTIP* and *Ft1* mRNA levels after LV-mediated RNAi. (A) *AKTIP* expression in HPFs at 7 dpi with ctr or different shAKTIP constructs (9, 10, 11, 12, and 13). (B) *Ft1* expression in p53^-/-^MEFs at 7 dpi with ctr or different shFt1 constructs (69, 70 and 73). The control vectors used in HPFs and MEFs contained a shRNA with no targets in human or mouse genome. RNA levels were measured by Q RT-PCR on total RNA extracts using gene-specific primers. Bars are the mean values ± SD of samples analyzed in duplicate. (C) Western blots from shAKTIP-11 infected (10 dpi) HPFs, HeLa and 293T cells, showing a strong reduction in AKTIP expression compared to mock control.
(TIF)

S2 Fig Characterization of DNA repair foci observed in AKTIP depleted HPFs. (A) Examples of DNA repair foci observed after X-irradiation (1 Gy) (B, C) Quantification of foci containing both γH2AX and 53BP1 (B) or both γH2AX and ATM-P (C), in HPFs infected (5 dpi) with shAKTIP-11, or irradiated (IR; X-ray, 1 Gy). 50 cells examined for each sample.
(TIF)

S3 Fig RNAi-mediated AKT downregulation. Western blotting shows reduced AKT expression in shAKT HPFs (10 dpi) compared to mock and ctr (10 dpi) controls.
(TIF)

S4 Fig AKTIP depletion does not induce abrupt telomere loss. (A) Frequency of chromatid ends lacking a FISH signal. Values are the means ± SD of two independent experiments; values from mock, ctr (7 dpi) and shAKTIP-11 (7 dpi) cells are not significantly different. (B) Southern blotting of HinfI/RsaI digested genomic DNA extracted from ctr- or shAKTIP-11-infected HPFs (13 dpi); telomeric DNA was detected with a TTAGGG repeat probe. Genomic DNA of late passage (LP, passage 30) untreated HPFs was used as control.
(TIF)

S5 Fig RNAi-mediated Ft1 and Trf1 downregulation. *Ft1* and *Trf1* mRNA levels after were determined at 7 dpi by Q RT-PCR on total RNA extracts using gene-specific primers. Bars are the mean values ± SD of samples analyzed in duplicate.
(TIF)

S6 Fig Comparison of the predicted AKTIP and Peo tridimensional models. (A) Alignment of the amino acid sequence of AKTIP, hUEV1, hUEV2, hUBC13 and Peo. Secondary structure elements predicted for AKTIP are shown above the alignment. Red and blue arrowheads indicate the sites of the catalytic Cys (Asp in AKTIP) and the HPN motif (HPL in AKTIP), respectively. The red dotted lines indicate predicted intrinsically disordered portions of AKTIP, and the blue dotted line the disordered region of Peo. (B) Comparison between the Peo and AKTIP models. The black arrows pointing outwards indicate the starting sites of the predicted disordered regions; AKTIP contains disordered regions of 70 and 60 aa at the N and C-termini, respectively; Peo only contains a disordered region of ~70% aa at its C terminus. These disordered regions are not represented in the tridimensional molecular models and are shown in the schematic linear models of the proteins. The variant Asp residues, and the HPL (AKTIP) and HPH (Peo) motifs are represented as sticks and indicated by red and purple arrows, respectively.
(TIF)

S1 Table Interfering sequences in lentiviral vectors. (DOCX)

S2 Table Primers for gene expression analysis. (DOC)

S3 Table Primers for cloning GST-tagged AKTIP fragments. (DOC)
