# Supplementary figures and images for: AKTIP/Ft1, a New Shelterin-Interacting Factor Required for Telomere Maintenance
Source: PLoS Genet. 2015 Jun 25;11(6):e1005167. doi: 10.1371/journal.pgen.1005167 (PMC4481533; doi:10.1371/journal.pgen.1005167)

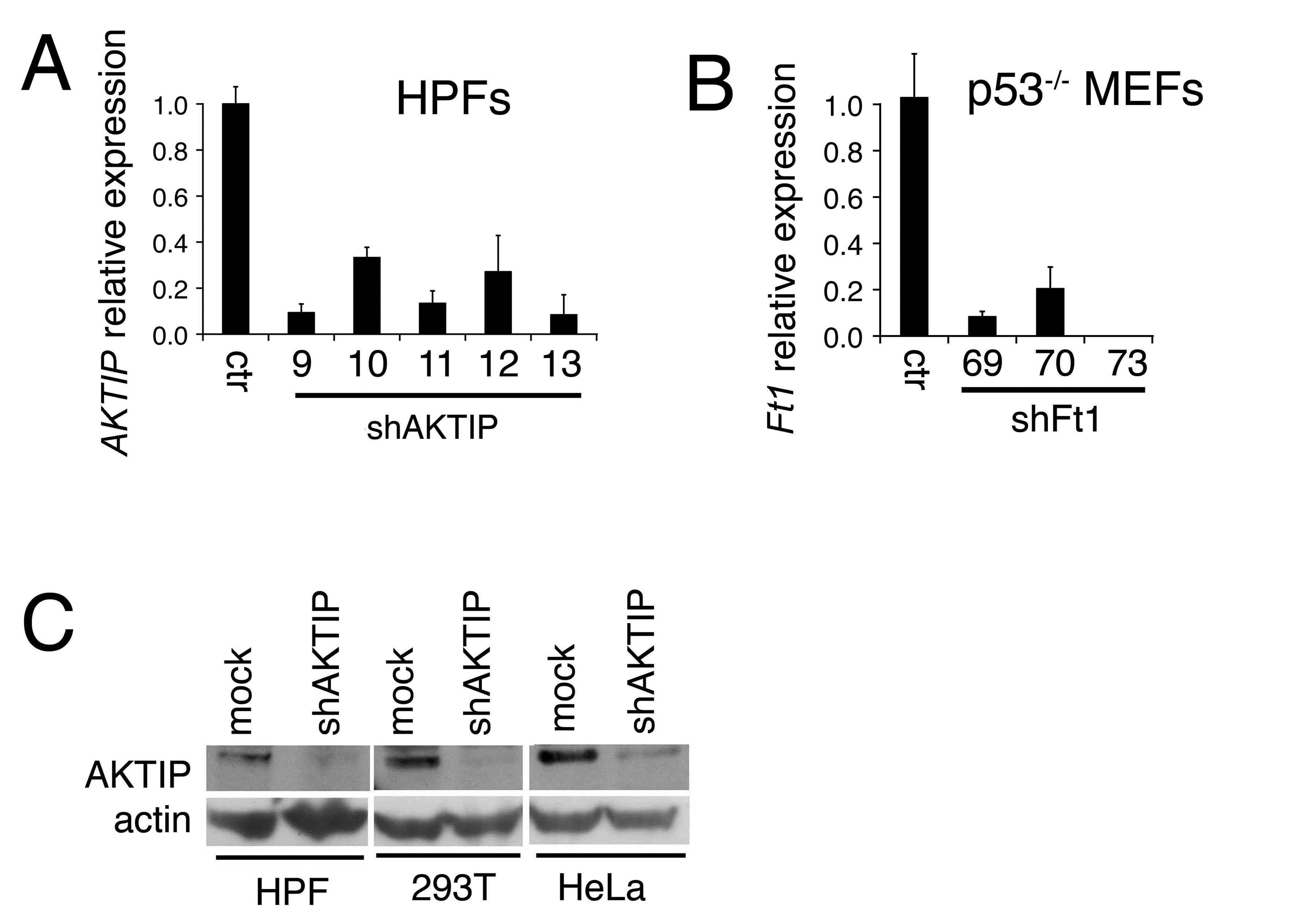

Supplement: S1 Fig — (A, B) AKTIP and Ft1 mRNA levels after LV-mediated RNAi. (A) AKTIP expression in HPFs at 7 dpi with ctr or different shAKTIP constructs (9, 10, 11, 12, and 13). (B) Ft1 expression in p53-/-MEFs at 7 dpi with ctr or different shFt1 constructs (69, 70 and 73). The control vectors used in HPFs and MEFs contained a shRNA with no targets in human or mouse genome. RNA levels were measured by Q RT-PCR on total RNA extracts using gene-specific primers. Bars are the mean values ± SD of samples analyzed in duplicate. (C) Western blots from shAKTIP-11 infected (10 dpi) HPFs, HeLa and 293T cells, showing a strong reduction in AKTIP expression compared to mock control. (TIF) [file pgen.1005167.s001.tif]

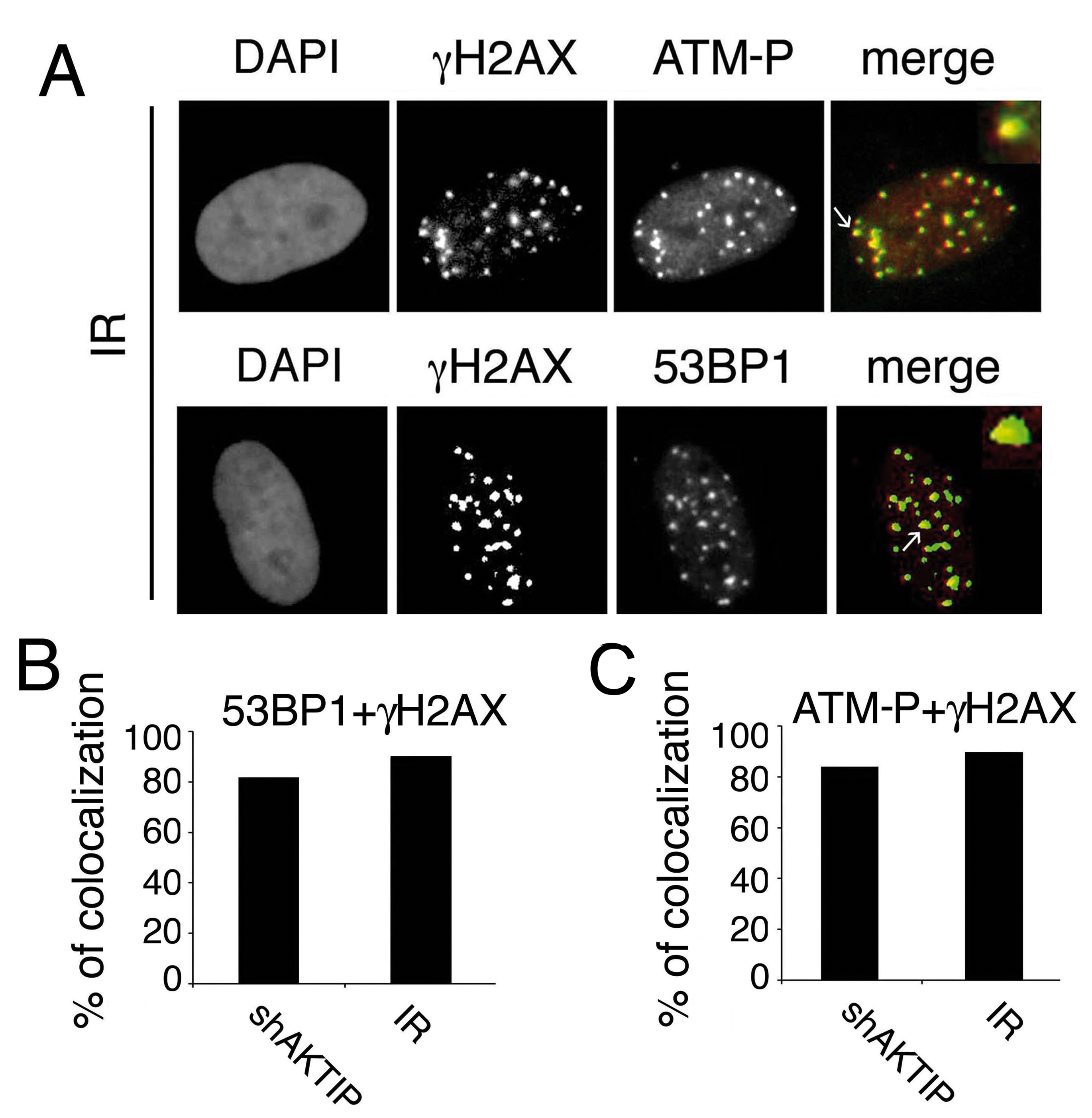

Supplement: S2 Fig — (A) Examples of DNA repair foci observed after X-irradiation (1 Gy) (B, C) Quantification of foci containing both γH2AX and 53BP1 (B) or both γH2AX and ATM-P (C), in HPFs infected (5 dpi) with shAKTIP-11, or irradiated (IR; X-ray, 1 Gy). 50 cells examined for each sample. (TIF) [file pgen.1005167.s002.tif]

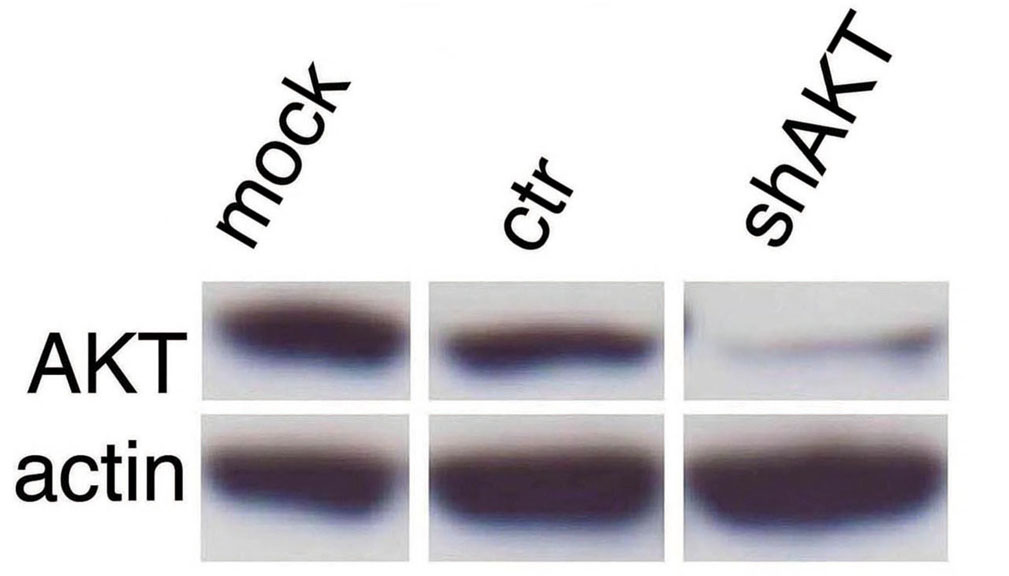

Supplement: S3 Fig — Western blotting shows reduced AKT expression in shAKT HPFs (10 dpi) compared to mock and ctr (10 dpi) controls. (TIF) [file pgen.1005167.s003.tif]

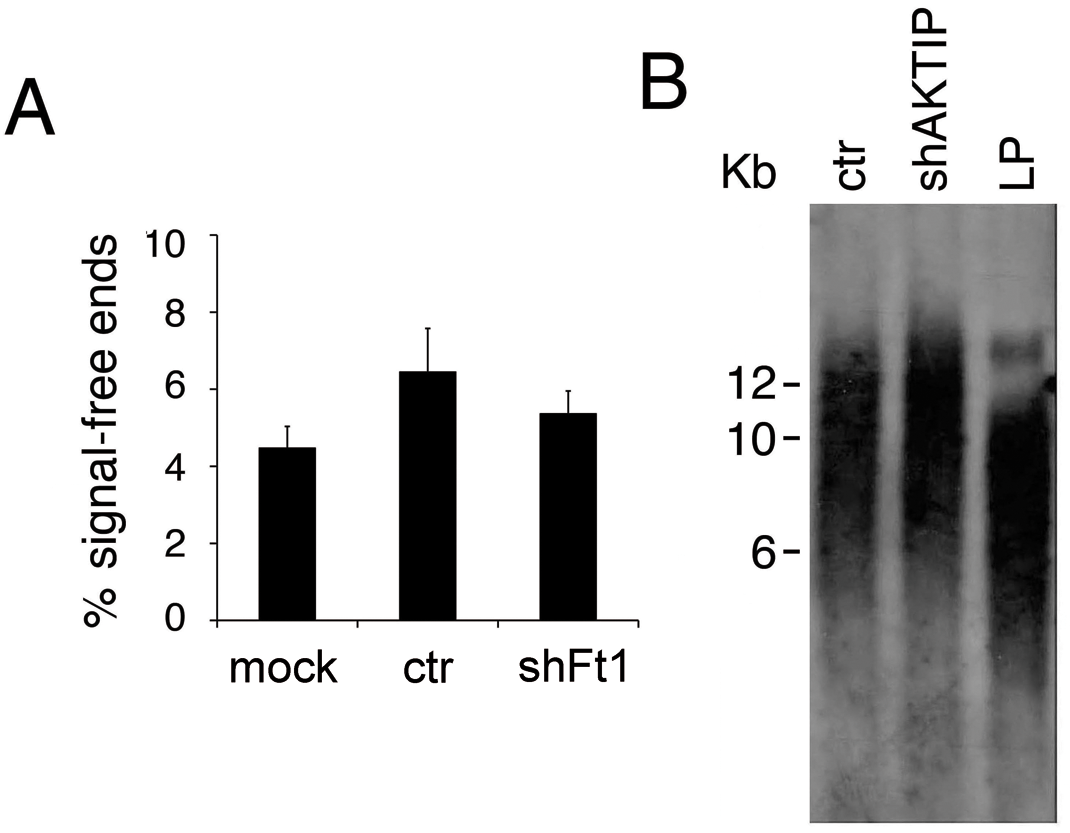

Supplement: S4 Fig — (A) Frequency of chromatid ends lacking a FISH signal. Values are the means ± SD of two independent experiments; values from mock, ctr (7 dpi) and shAKTIP-11 (7 dpi) cells are not significantly different. (B) Southern blotting of HinfI/RsaI digested genomic DNA extracted from ctr- or shAKTIP-11-infected HPFs (13 dpi); telomeric DNA was detected with a TTAGGG repeat probe. Genomic DNA of late passage (LP, passage 30) untreated HPFs was used as control. (TIF) [file pgen.1005167.s004.tif]

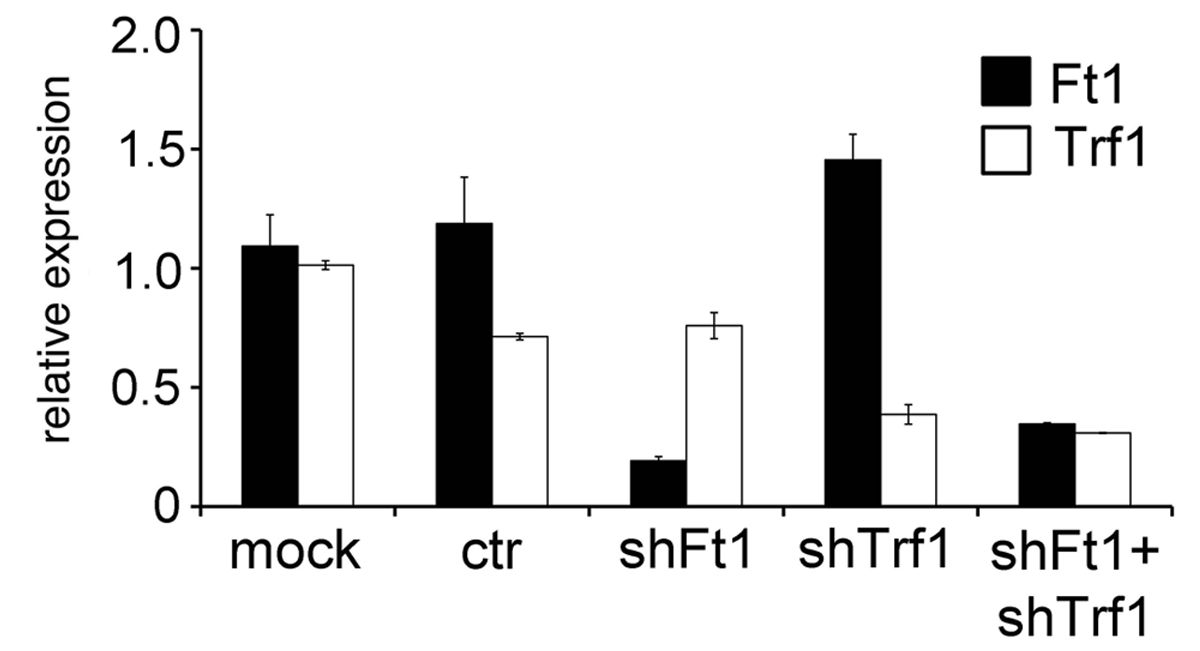

Supplement: S5 Fig — Ft1 and Trf1 mRNA levels after were determined at 7 dpi by Q RT-PCR on total RNA extracts using gene-specific primers. Bars are the mean values ± SD of samples analyzed in duplicate. (TIF) [file pgen.1005167.s005.tif]

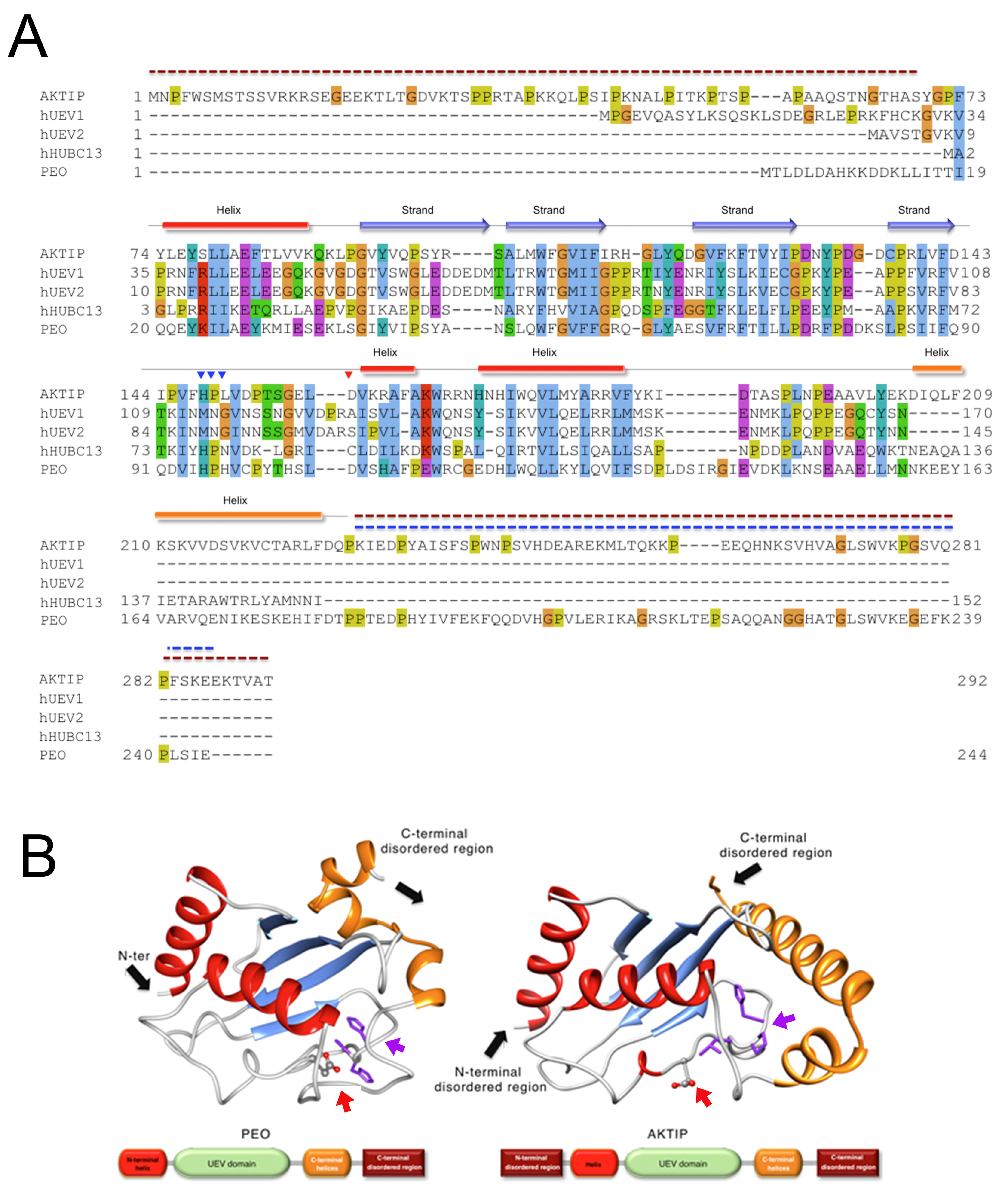

Supplement: S6 Fig — (A) Alignment of the amino acid sequence of AKTIP, hUEV1, hUEV2, hUBC13 and Peo. Secondary structure elements predicted for AKTIP are shown above the alignment. Red and blue arrowheads indicate the sites of the catalytic Cys (Asp in AKTIP) and the HPN motif (HPL in AKTIP), respectively. The red dotted lines indicate predicted intrinsically disordered portions of AKTIP, and the blue dotted line the disordered region of Peo. (B) Comparison between the Peo and AKTIP models. The black arrows pointing outwards indicate the starting sites of the predicted disordered regions; AKTIP contains disordered regions of 70 and 60 aa at the N and C-termini, respectively; Peo only contains a disordered region of ~70% aa at its C terminus. These disordered regions are not represented in the tridimensional molecular models and are shown in the schematic linear models of the proteins. The variant Asp residues, and the HPL (AKTIP) and HPH (Peo) motifs are represented as sticks and indicated by red and purple arrows, respectively. (TIF) [file pgen.1005167.s006.tif]
