# Supplementary material for: AKTIP/Ft1, a New Shelterin-Interacting Factor Required for Telomere Maintenance
Source: PLoS Genet. 2015 Jun 25;11(6):e1005167. doi: 10.1371/journal.pgen.1005167 (PMC4481533; doi:10.1371/journal.pgen.1005167)
Supplement: S1 Table — (DOCX) [file pgen.1005167.s007.docx]

**Supplemental Table 1: Interfering sequences in lentiviral vectors**

| *Sh sequence name* | *5’-3’ sh sense sequence* | *Target gene*  *(Gene ID)* | *Target position* |
| --- | --- | --- | --- |
| Shctr | CAACAAGATGAAGAGCACCAA | none | none |
| ShAKTIP 09 | TGAAGGTGAAGAGAAGACATT | *AKTIP*  (64400) | CDS  (258-279) |
| ShAKTIP 10 | TCCACGAACTGCACCAAAGAA | *AKTIP*  (64400) | CDS  (303-324) |
| ShAKTIP 11 | CACTGCTCGTTTGTTTGACCA | *AKTIP*  (64400) | CDS  (870-891) |
| ShAKTIP 12 | TGCCCATAACTAAGCCTACAT | *AKTIP*  (64400) | CDS  (353-374) |
| ShAKTIP 13 | CCTGTCTCTAAGTAATGCATT | *AKTIP*  (64400) | 3’UTR |
| ShAKT | CGCGTGACCATGAACGAGTTT | *AKT1*  (207) | CDS  (769-790) |
| ShFt1 69 | GCTGTTTGATATTCCCGTCTT | *Ft1*  (14339) | CDS  (632-653) |
| ShFt1 70 | CCTTTCAGTAAAGAAGAGAAA | *Ft1*  (14339) | CDS  (1056-1077) |
| ShFt1 73 | CCTGATGAACAGCACAATAAA | *Ft1*  (14339) | CDS  (987-1008) |
| shTrf1 938 | GAACGCCTTATCGCAGTTAAA | *Trf1*  (21749) | CDS  (938-959) |
