# Supplementary material for: AKTIP/Ft1, a New Shelterin-Interacting Factor Required for Telomere Maintenance
Source: PLoS Genet. 2015 Jun 25;11(6):e1005167. doi: 10.1371/journal.pgen.1005167 (PMC4481533; doi:10.1371/journal.pgen.1005167)
Supplement: S2 Table — (DOC) [file pgen.1005167.s008.doc]

Supplemental Table 2: Primers for gene expression analysis

| *Target gene* | *5’-3’ oligonucleotide sequence* |
| --- | --- |
| *AKTIP* Forward | TCCACGCTTGGTGTTCGAT |
| *AKTIP* Reverse | TCACCTGAGGTGGGATCAACT |
| *Ft1* Forward | CCGTCTTTCACCCACTAGTTGAT |
| *Ft1* Reverse | TTGCGAACGCTCTTTTCACA |
| *mGAPDH* Forward | GTGGCAAAGTGGAGATTGTTGCC |
| *mGAPDH* Reverse | GATGATGACCCGTTTGGCTCC |
| *GAPDH* Forward | TGGGCTACACTGAGCACCAG |
| *GAPDH* Reverse | GGGTGTCGCTGTTGAAGTCA |
| *p21* Forward | TGGAGACTCTCAGGGTCGAAA |
| *p21* Reverse | GGCGTTTGGAGTGGTAGAAATC |
| *Trf1* Forward | ACAGACGTGCTCCATCAGATT |
| *Trf1* Reverse | CCTTCCACTGGTTCTTCGGT |
