# Supplementary material for: AKTIP/Ft1, a New Shelterin-Interacting Factor Required for Telomere Maintenance
Source: PLoS Genet. 2015 Jun 25;11(6):e1005167. doi: 10.1371/journal.pgen.1005167 (PMC4481533; doi:10.1371/journal.pgen.1005167)
Supplement: S3 Table — (DOC) [file pgen.1005167.s009.doc]

**Supplemental Table 3: Primers for cloning GST-tagged *AKTIP* fragments**

| *Targetd AKTIP amino acid (aa)* | *5’-3’ oligonucleotide sequence* |
| --- | --- |
| AKTIP aa1 Forward | agtgacgggGTCGACcATGAACCCTTTCTGGAGC |
| AKTIP aa70 Forward | agtgacgggGTCGACcTATGGACCCTCCTACCTG |
| AKTIP aa102 Forward | agtgacgGGATTCggGTCGACcTCTGCATTAATGTGGTTT |
| AKTIP aa190 Forward | agtgacgGGATTCggGTCGACcACAGCAAGCCCCCTGAAC |
| AKTIP aa70 Reverse | agtgacgGCGGCCGCGAATTCCTAATAGGACGCATGCGTGCC |
| AKTIP aa101 Reverse | agtgacgGCGGCCGCGAATTCCTAGCGATAAGATGGCTGCAC |
| AKTIP aa189 Reverse | agtgacgGCGGCCGCCTAATCAATCTTGTAGAAAAC |
| AKTIP aa232 Reverse | agtgacgGCGGCCGCCTAGTCTTCTATTTTAGGTTG |
| AKTIP aa292 Reverse | agtgacgGCGGCCGCTCAAGTCGCCACTGTTTTCTCTTC |
